# Transcriptomes of testis and pituitary from male Nile tilapia (*O*. *niloticus* L.) in the context of social status

**DOI:** 10.1371/journal.pone.0268140

**Published:** 2022-05-11

**Authors:** Michelle Thönnes, Rebecca Prause, Berta Levavi-Sivan, Frank Pfennig

**Affiliations:** 1 Environmental Monitoring and Endocrinology, Faculty of Biology, Technische Universität Dresden, Dresden, Germany; 2 Department of Animal Sciences, The Robert H. Smith Faculty of Agriculture, Food, and Environment, Hebrew University of Jerusalem, Rehovot, Israel; Universita degli Studi di Bari Aldo Moro, ITALY

## Abstract

African cichlids are well established models for studying social hierarchies in teleosts and elucidating the effects social dominance has on gene expression. Ascension in the social hierarchy has been found to increase plasma levels of steroid hormones, follicle stimulating hormone (Fsh) and luteinizing hormone (Lh) as well as gonadosomatic index (GSI). Furthermore, the expression of genes related to gonadotropins and steroidogenesis and signaling along the brain-pituitary-gonad axis (BPG-axis) is affected by changes of an animal’s social status. In this study, we use RNA-sequencing to obtain an in-depth look at the transcriptomes of testes and pituitaries from dominant and subordinate male Nile tilapia living in long-term stable social hierarchies. This allows us to draw conclusions about factors along the brain-pituitary-gonad axis that are involved in maintaining dominance over weeks or even months. We identify a number of genes that are differentially regulated between dominant and subordinate males and show that in high-ranking fish this subset of genes is generally upregulated. Genes differentially expressed between the two social groups comprise growth factors, related binding proteins and receptors, components of Wnt-, Tgfβ- and retinoic acid-signaling pathway, gonadotropin signaling and steroidogenesis pathways. The latter is backed up by elevated levels of 11-ketotestosterone, testosterone and estradiol in dominant males. Luteinizing hormone (Lh) is found in higher concentration in the plasma of long-term dominant males than in subordinate animals. Our results both strengthen the existing models and propose new candidates for functional studies to expand our understanding of social phenomena in teleost fish.

## Introduction

Reproduction requires the coordination of gonad development and behavior subject to environmental condition and available resources. Embryonic gonad development, onset of puberty, seasonal changes in gonad structure or social effects on fertility are controlled by the endocrine system along the brain-pituitary-gonad axis (BPG-axis) in all vertebrates. Thus, animals can respond adequately to environmental influences. Intricate signaling pathways synchronize social cues, production of steroid hormones, growth factors, gametogenesis and finally successful reproduction. The canonical BPG-axis signaling hierarchy begins with hypothalamic neurons secreting gonadotropin releasing hormone (Gnrh) which stimulates the release of gonadotropins (Gth) in the pituitary. Fish possess two Gths, follicle stimulating hormone (Fsh) and luteinizing hormone (Lh). Gths are heterodimeric hormones that consist of a common α-subunit and a hormone specific β-subunit. In recent years, genes encoding Gth subunits have been isolated from over 50 fish species. Like in other vertebrates, gonadotropin releasing cells (gonadotropes) of teleosts form organized structures in the pituitary. While most tetrapod gonadotropes secrete both Gths, in teleosts Lh and Fsh are produced in distinct cells forming their own functional networks [1–3]. The steroid hormones such as testosterone and estradiol are significantly involved in the passing on of gonadotropic signals at the level of the gonads in vertebrates. In many male teleosts, even more than testosterone itself, the testosterone derivative 11-KT plays a central role in the control of gonad development and spermatogenesis [4, 5].

It has now been known for decades that steroid hormones are essential for relaying gonadal feedback to the brain and pituitary [6]. Since then, it has been established that neurosteroids can also be produced in the teleost brain [7, 8]. Aside from steroid hormones, a variety transcription and growth factors have been discovered to influence the BPG-axis in teleost fish, as summarized by Rajakumar and Senthilkumaran 2020 [9] or Zohar *et al*. [10].

Much research effort is directed to elucidate gonad development and differential gene expression between males and females, as well as the process of sex determination [4, 11, 12]. In this study however, we want to focus on fish of the same sex but with different social status. The Nile tilapia *Oreochromis niloticus* is a practical model for such an undertaking because this species establishes stable, long-term hierarchies [13]. *O*. *niloticus* belongs to the African cichlids, which are known for their territorial behavior and the establishment of social hierarchies [14]. For group living animals, social interactions are important factors influencing their physiological state and their own behavior. 11-ketotestosterone (11-KT) and cortisol levels of *O*. *mossambicus* for example change depending on the presence of conspecifics and also with the social context [15]. Very comprehensive studies about social dynamics have been conducted on *Astatotilapia burtoni*. Like *O*. *niloticus* it is an African cichlid with a lek-like mating system that exhibit parental caring (mouthbrooding). In both species, males establish hierarchies, which are characterized by aggressive, territorial behavior and different colorations of dominant and subordinate males. Male cichlids can change their hierarchy status in a matter of minutes. Both social ascend [14, 16] and descend [17] can cause rapid changes in androgen and stress hormone levels.

For such rapid changes to occur, subordinate males have to maintain a certain level of spermatogenic capacity [18] and in Nile tilapia complete testicular degeneration is an exception in subordinate males [13]. In contrast to previous studies mentioned above, we do not focus on the transition period (descend, ascend) but look at well-established social hierarchies. Here, we study differences in male testis and pituitary transcriptomes via RNA sequencing. In order to identify the factors most crucial for dominance, our study comprises sexually mature animals of different ages and with long phases of continuous dominance. With the transcriptome approach, we want to emphasize the processes involved in establishing and maintaining social status in male Nile tilapia. This will allow us to identify new factors affecting the BPG-axis in this specific aspect of tilapia reproduction.

## Methods

### Fish maintenance and sampling

*Oreochromis niloticus* L. for this study were bread and reared at the institute’s animal facility in Dresden since 2004. All specimens originated from a brood stock from the Leibniz-Institut für Gewässerökologie und Binnenfischerei Berlin, Germany. Purebred *Oreochromis niloticus* came in 1994 from the Research Institute of Fish culture and Hydrobiology in Vodnany, Czech Republic, to Berlin (personal communication Bernhard Rennert, Leibniz-Institut für Gewässerökologie und Binnenfischerei Berlin). The animals originate from a catch of around 60 fries from Sudan in 1985, on which the later brood stock was based. In 1986, nine of these individuals founded the Nile tilapia production with heated effluent water from thermal power plant in Tisova (personal communication Dr. Zdenek Adamek, Institute of Vertebrate Biology, Czech Academy of Sciences, Brno, Czech Republic). In our facility, the fish were kept in 550 L or 820 L tanks at 26 °C and a 14 h light: 10 h dark photoperiod. In the recirculating systems, five percent of the water was changed every day. Electric conductivity was kept around 600 μS. Fish were fed *ad libitum* twice a day with commercially available food (Pro Aqua Brut 1.0 MP, Skretting, Norway) for the first six months. Afterwards their diet was changed to F3-P Optiline (6mm, Skretting, Norway). From then on, the animals were fed *ad libitum* once per day. Vitamins (multivit, hw-Wiegand, Germany) and trace elements (tracevit, hw-Wiegand, Germany) were supplemented once per week and once per month, respectively.

Fish were housed in family groups of 20–30 siblings from the same brood. A plastic tube was placed in every tank as shelter/spawning site. When fish reach sexual maturity around six months, males typically start to fight over the tube which serves as habitat enrichment and the bottom area of the tank. As soon as a single male succeeds in claiming the dominant rank, no other fish are tolerated to approach the tube, except for females during mating. Henceforth, males can be divided into dominant males (d-males) and subordinate males (s-males).

Experimental animals were anaesthetized with benzocaine 0.2 g/L. Blood was drawn from the caudal vein with a heparinized needle and transferred into a tube containing 10 μl of heparin (1000 IU/ml). Plasma from 1–2 ml blood was separated by centrifugation before snap freezing in liquid nitrogen and storing at -80 °C. Fish were killed by severing of the spinal cord. All experimental animals were weighted and measured. The testes were excised and weighted in order to calculate the GSI (gonadosomatic index = gonad weight/total weight *100). From the middle section of each testis strand, slices were cut for RNA extraction and histology.

Animal culture and euthanasia were performed according to the EU directive 2010/63/EU guidelines as well as to the German national regulations and animal welfare (Tierschutzgesetz §11, Abs. 1, no. 1). Fish husbandry and procedures are approved by administrative regulations (Landesdirektion Sachsen, File number 25-5131/346/6). This approval process by the Landesdirektion Sachsen requires prior examination by the Technische Universität Dresden Animal Care and Use Committee (TU Dresden IACUC). All procedures and experiments of our research was discussed and approved by the TU Dresden IACUC (authorization number 25-5131/525/2).

### Hormone measurements

11-Ketotestosterone (11-KT) concentrations in the plasma were measured with the 11-KT ELISA kit from Cayman Chemical (USA). Before the actual testing, the samples were extracted with ethyl acetate/hexane as described in the manufacturer’s instructions. For each plate a standard curve was run, using serial dilutions of the bulk standard included in the kit. All samples were measured in two dilutions and the assay was run in triplicate. The dilutions were chosen according to the status of the animals: 1:500 and 1:200 for d-males, 1:200 and 1:100 for s-males and 1:100 and 1:50 for females. Lh and Fsh plasma levels of animals chosen for RNA-Seq were measured with specific competitive ELISAs based on recombinant gonadotropins that were created by using the yeast *Pichia pastoris* [19, 20]. Sensitivity for the plasma measurements were 15.84 pg/ml for Lh, 0.24 pg/ml for Fsh and 30.0 pg/ml for Gh. Inter-assay coefficient of variation (CV) was 14.8, 12.5, and 13.0%, while intra-assay CV was 7.2, 8.0, and 8.0% for Lh, Fsh and Gh, respectively. All these hormone measurements were performed for all eight animals evaluated by RNA-sequencing.

Testosterone (T) and 17β-estradiol (E2) levels were measured with ELISA kits from IBL (Germany). Two plasma samples (9S and 18D) could not be tested for these two hormones due to limited samples. Steroid extraction and concentration were performed for 17β-estradiol ELISA. For testosterone ELISAs no extraction was performed and samples were measured in two dilutions (1:5 and 1:20). Standard curves for the IBL kits were calculated with CurveExpert 1.4 [21]. % CV was calculated as intra-essay control for all ELSIA experiments. Maximum % CV observed was 7.5%.

### Choosing experimental animals for RNA-Seq

Dominant fish were identified by observation at least four weeks before the experiment. Males which claim the top position of the hierarchy can be unambiguously identified by their pale body coloration, white irises and aggressive, territorial behavior as described above. When a female becomes ready to spawn, dominant males also display typical courtship behavior. Subordinate males on the other hand are similar to females in their coloration and appearance, but usually bigger. The shape of the anal papilla was used to discriminate between subordinate males and females. Striking bodily features such as fin shape and size were used to check the animals’ social status twice every week. The animals from one family with corresponding dominant-subordinate males were removed from the tank at the same time for sampling.

When choosing suitable samples for transcriptome analysis we resorted to several criteria to identify dominant and subordinate males. To define a dominant-subordinate pair, the d-male’s GSI had to be twice as high as that of his subordinate counterpart. The GSI of the latter however should not be below 0.05 to exclude rare cases of gonadal degeneration. Histology was applied to confirm normal testis development. Finally, the 11-KT concentration in the blood was consulted as a measure of dominance. By the definition used in this study, d-males had 11-KT concentrations above 4 ng/ml. Furthermore, it had to be at least twice as high as in the corresponding s-males. Only when all criteria described above were fulfilled fish were chosen for RNA-sequencing. Thus, we chose three dominant males from different families and one or two matching subordinate males from the same families. In this way we generated samples from a total of eight animals. Fish were 9 months, 12.5 months or 34 months of age. For the RT-qPCR experiment we used additional four fish (two dominant and two subordinate males) to the already existing eight samples from RNA-sequencing in order to calculate more robust statistics.

### RNA extraction, quality control and quantification

Fresh tissue slices (approx. 50 mg) were snap frozen in liquid nitrogen and stored at -80 °C until further processing. Total RNA was extracted from the organ pieces after cultivation using peqGOLD TriFast (PEQLAB-Lifescience, Germany) according to the manufacturer’s protocol. The organ pieces were macerated by hand with micro-pestles (USA Scientific, USA) before extraction. RNA quantification and quality control were performed on a 2100 Bioanalyzer (Agilent, USA) at the Max-Planck Institute of molecular cell biology and genetics, Dresden (Germany). RIN values were between 6.5 and 9.1 (Table 1). For RT-qPCR validation of RNA-Seq results, RNA quality was assessed on a 2% agarose gel and by a Nanodrop 1000 spectrometer (Thermo Fischer Scientific, USA) device. To prepare samples for downstream applications, they were treated with DNase I (recombinant, RNase free from Roche, Switzerland) to digest any contaminating gDNA.

**Table 1:** RIN values of RNA from testes and pituitaries.

Tissue	ID	RIN	Tissue	ID	RIN
testis	7S	9.0	pituitary	7S	9.2
	9S	8.2		9S	9.0
	8D	9.1		8D	9.0
	17S	8.8		17S	8.4
	18D	8.7		18D	8.9
	30S	6.5		30S	8.5
	31S	7.5		31S	8.1
	32D	6.5		32D	8.7

RNA quality was measured with Agilent 2100 Bioanalyzer. RIN values can vary between 10 (undegraded) and 0 (completely degraded).

### cDNA synthesis and RT-qPCR

RNA-Seq results were validated by RT-qPCR of 15 genes. This test was performed on testis RNA from a total of 12 animals, all eight fish chosen for RNA-Seq and an additional four fish (two s-males and two d-males which had maintained their rank for 6 months). The surplus samples were added to obtain more robust statistics. RNA extraction for sequencing and validation were performed on parallel tissue samples on different days. Total RNA (2–4 μg) was reverse transcribed into cDNA using the High-Capacity Reverse Transcription Kit (ThermoFisher Scientific, USA) with random hexamers according to the manufacturer’s protocol. Successful cDNA synthesis and DNase I treatment were validated with PCR for the β-actin gene (*actb*). The *actb* primers binding sites are separated by an intron, allowing discrimination between gDNA and cDNA products (Table 2).

**Table 2:** Primers for RT-qPCR and PCR.

Task	Gene	Ensembl ID	Forward	Reverse	Fragment size (bp) on cDNA	Efficiency (%)	Source
RT-qPCR	*acvr2bb*	ENSONIG00000018510	TGCTCCGACACAGATGGAAC	TGGTCTCACACATGTGGCTC	172	103.3	this work
	*amh*	ENSONIG00000004781	AACGTACGACGCGCAAAG	TCTCGATGTGGGAGTTGAGC	215	108.9	[13]
	*amhR*	ENSONIG00000012464	GTGTATTCAACTGGGCACGC	GCAGTTCCCAGTGTTCAGGT	284	94.4	this work
	*ar*	ENSONIG00000012854	GGCCTAATTACAGCTTCGGC	ATAAGGCGTCCTCAGCATCC	170	127.5	this work
	*CTNNB1*	ENSONIG00000002079	ACGCTATCGTTGGTGGTCTT	ACGGACATCATTTGACGATGGTA	230	100.7	this work
	*ctnnb2*	ENSONIG00000007226	ACCGTTCTTTCCATTCGGGG	AACCAGGCCAGTTGGTTTGA	190	98.6	this work
	*cyp11c1*	ENSONIG00000006462	CAAAGAAGTCCTCAGGTTGTACCCA	GGACCAAAGTTCCAGCAGGTATGT	103	105.7	[22]
	*dkk3b*	ENSONIG00000010975	GTATCCCGACAGACATGCCC	CAGGTTGGGGTGGCTTAGAC	230	98.5	this work
	*fshr*	ENSONIG00000015917	CTGAGAACGACCTGCTGGAG	GCCCCGATATTCCTCAGAGC	106	107.1	[23]
	*gthrII*	ENSONIG00000009844	AGCTGGAATATTTGAGCATCTCT	AGCGTGTTGAGCTTAGTCCC	235	109.5	this work
	*hsd11b2*	ENSONIG00000003266	AAGAGACAATGATGACACAGG	GCTACATTTTGGTTCTTGACAG	170	107.6	[24]
	*igf3*	ENSONIG00000017948	CAGACACTCCAGGTGCTGTGTG	CAAGCCTTTACGTAAATAGATTCC	168	100.9	[25]
	*inha*	ENSONIG00000013357	GGAATGTCTTGACAGATGCCT	CAGGGGAATGTGTCTCCGTC	167	112.1	this work
	*insl3*	ENSONIG00000031714	AAGCTGCACGTTTACAGTGAT	TTTTTCCAGCAGTTCGCCTG	193	108.9	this work
	*stAR2*	ENSONIG00000010793	GTTTGAGGTTGCTGAGAGTAATGGT	TTGCTGTATGCTTGGGTTCC	169	107.3	[26]
	*ef1α*	ENSONIG00000035055	TCCGTCGTGGATACGTTGC	ATGTGGGCAGTGTGGCAATC	145	104.6	this work
	*gapdh*	ENSONIG00000012916	AAGGGTGGTGCCAAGAGA	GCAGTTGGTTGTGCAGGA	132	106.2	this work
	*dmrt1*	ENSONIG00000014201	CGGCCCAGGTTGCTCTGAG	AACGTGAAGACAGCGTGAAG	139	96.5	F: [27], R: [28]
PCR	*actb*	ENSONIG00000003145	GATCCGGTATGTGCAAGG	CTTCTCCCTGTTGGCTTTGG	cDNA:317, gDNA:519		[28]

Quantitative real-time PCR was performed using a QuantStudio 5 Real-Time PCR System (Thermo Fisher Scientific, USA). The reactions were carried out in a total volume of 10 μl, containing 5 μl PowerUp SYBR Green Mastermix (Thermo Fisher Scientific, USA), final primer concentrations of 250 nM, and 22.5 ng cDNA per well. The reaction was run in triplicate for each sample. The cycler was used with the following setting:

50 °C for 2 min, 95 °C for 10 minutes, followed by 40 cycles of 95 °C for 15 sec and 60 °C for 1 min, and a final dissociation step at 95 °C for 1 min. A no template control (NTC) was always included for each primer set. Standard curves with serially diluted cDNA were used to check primer efficiencies (Table 2). The gene expression was calculated as described in Taylor *et al*. [29]. Gene expression was normalized to the reference genes *ef1*, *dmrt1* and *gapdh*. Reference gene stability was tested with BestKeeper [30] (also compare Table 3). All primers used for RT-qPCR are listed in Table 2.

**Table 3:** Housekeeping gene evaluation with BestKeeper.

Regression Analysis				
Statistic	BestKeeper	*ef1*	*gapdh*	*dmrt1*
n	12	12	12	12
geo Mean [CP]	21.59	19.09	23.43	22.5
ar Mean [CP]	21.6	19.1	23.44	22.51
min [CP]	20.51	18.01	21.94	21.51
max [CP]	22.78	20.38	24.86	23.32
std dev [± CP]	0.56	0.57	0.64	0.51
CV [% CP]	2.6	3	2.75	2.26
min [x-fold]		-2.17	-2.93	-2.08
max [x-fold]		2.54	2.81	1.84
std dev [± x-fold]		1.51	1.59	1.44

### RNA-sequencing and DEG retrieval

Tissue samples were taken from testes and pituitaries of the experimental animals for RNA preparation. The RNA was sequenced at the Deep Sequencing Facility of the CMCB technology platform (Dresden, Germany). For mRNA libraries 300 ng of total RNA were used. Poly-dT pulldown was performed to enrich mRNA in the samples. Libraries with sizes between 300 bp and 350 bp were prepared with the NEBNext^®^ Ultra^™^ II Directional RNA Library Prep Kit (Illumina, USA) and amplified for 12 PCR cycles. The single end sequencing itself was carried out on a HiSeq2000 Next Generation Sequencer (Illumina, USA) with a read length of 75 bp. Between 2.8x10^7^ and 3.5x10^7^ reads per sample were obtained in this way. Trimming of undetermined and low-quality bases as well as of the Bioo Scientific Next FLEX 3’ adapter was done with cutadapt (v1.8.1) [31] and the following parameters: “—trim-n -m 18 -q 10 -O 12 -a truseq_seqmatic_nextflex_5p_1 = TGGAATTCTCGGGTGCCAAGG -n 2—discard-untrimmed -a truseq_seqmatic_nextflex_5p_2 = TGGAATTCTCGG”.

Coverage analysis was done with BEDTools 2.26.0 [32]. Over 93% of the reads could be uniquely aligned to the reference (Orenil1.0 ensemble93) with BWA 0.7.10 [33] and GSNAP: 2018-05-30 [34]. Quality control on the bam files was performed with RNA-SeQC 1.1.8 [35]. Samtools 1.4.0 [36] was used for filtering of the files and read summarization was done with featureCounts 1.6.2 [37].

Normalization of raw counts based on library size and testing for differential expression between the different cell types/treatments was done with the DESeq R package (v1.20.0) [38]. Sample deviation from the gene average and PCA of the top 500 genes based were computed based on normalized and log2-transformed counts. To identify differentially expressed genes, counts were fitted to the negative binomial distribution and genes were tested between conditions using the Wald test of DESeq2 including age as a covariate. Resulting p-values were corrected for the multiple testing problem with Benjamini-Hochberg. Genes with a maximum of 5% false discovery rate (padj ≤ 0.05) were considered as significantly differentially expressed. Expression of 2 TPM (transcripts per million) was set as a cut-off for DEGs in order to filter for genes with a meaningful contribution to the observed differences between dominant and subordinate males.

In order to evaluate expression of genes not annotated in the genome version Orenil1.0 ensemble93, fastq sequences were mapped to the current genome release O_niloticus_UMD_NMBU with bowtie2 [39]. Gene quantification was done with RSEM [40] and DEGs were calculated with DESeq2 [38] as described above. Age correction was again applied. The fastq files were uploaded to the NCBI Sequence Read Archive (SRA) under the BioProject ID PRJNA817031.

### Functional annotation

Functional annotation and pathway enrichment analysis were performed with the DAVID 6.8 tool [41, 42]. Missing annotations of DEGs were added manually. Manual annotations were based on the latest Nile tilapia genome version (O_niloticus_UMD_NMBU) in Ensembl [43] or NCBI BLAST^®^ (megablast, standard settings) [44]. The gene lists (S1 Table) for the analysis were prepared as follows: 76 long noncoding RNAs were removed from the testis dataset. 92% (1314) genes were recognized by DAVID via their ENSONIG identifier. Additional 78 genes could be identified via LOC identifier or official gene symbol. Another 35 genes were not found in DAVID’s *O*. *niloticus* database but in other teleosts (*Danio rerio*, *Poecilia latipinna* and *Oryzias latipes*) and for one gene (*oct2*) the closest reference was found in chicken (*Gallus gallus*) only. Thus, an entry file containing 1428 DEGs in Entrez Gene ID format could be used for the analysis.

From the 463 originally identified DEGs in the pituitary, 33 were long noncoding RNAs. 365 of the remaining ENSONIG identifiers were promptly recognized by DAVID. 38 genes could be added to the list via their LOC identifiers or official gene symbols. Including *D*. *rerio* homologues yielded another 16 genes. Taken together, the final list for functional annotation comprised 419 IDs.

Gene lists were submitted to DAVID with the built-in *Oreochromis niloticus* background. Gene lists comprised DEGs with an adjusted p-values < 0.05. UP-Keywords (functional categories) and GO-terms (gene ontology) were calculated with standard parameters. Only results with Bonferroni corrected p-values <0.5 were considered sufficiently robust for further evaluation.

### Histology

Testis pieces of 5 mm were fixated for 30 min in 2% PFA/ HEPES (100 mM) at RT. Then, they were stored in 4% PFA/HEPES at 4 °C over night. Afterwards, organ slices were kept in 1% PFA/HEPES at 4 °C until use. Before vibratome sectioning, the testis fragments were washed in HEPES (100 mM) and embedded in 3% agarose blocks. These blocks were cut with a Hyrax V50 vibratome (Zeiss, Germany) with the following settings: slice thickness 200–250 μm, amplitude 0.3, trimming 40, frequency 60 Hz, blade angle 3 °. Until further processing, the slices were stored in 1% PFA/HEPES at 4 °C. Confocal laser scanning microscopy allowed analyzing different optical slices from the same testis slices. Two to four peripheral regions per testis slice were randomly chosen for recording z-stacks of 30 to 60 μm in depth by confocal microscopy (AxioObserver LSM880, Zeiss, Germany). Based on a previous study [13], we expected social status to be reflected by the thickness of the tunica albuginea (TA) and thus we focused on the peripheral regions of the testis slices. Measurements were taken on four to six images per z-stacks. The sections were treated with Draq5^®^ (biostatus, UK, 5 μM f.c.) to stain the cell nuclei. Under the confocal microscope, cellular composition of the testis, especially the nuclear morphology, becomes clearly visible. Classification of male germ line cells is based on the measurement of the nuclear diameter together with the number of cells per spermatogenic cyst according to the values given by Schulz et al. [45]. Deviating from the classification of Schulz et al., we have combined different generations of the different germ cell stages into larger groups. This results in the following staging of germ line cells in the present work in comparison to the classification given by Schulz et al. [45]: type A spermatogonia (largest and single germ cells with nuclear diameter larger than 8 μm) = primary spermatogonia; type B spermatogonia (nuclear diameter 5–8 μm, in cysts with 2 to 128 cells) = secondary spermatogonia from generation B1 until B7; primary spermatocytes (nuclear diameter 4,6–6,5 μm, in cysts with about 200 cells and at different stages of meiosis I from preleptotene, leptotene/zygotene, pachytene until diplotene spermatocytes) = primary spermatocytes; secondary spermatocytes (nuclear diameter about 4,3 μm, roughly 400 cells per cyst) = secondary spermatocytes; spermatids (2–3,4 μm, smallest germ line cells and highest number of cells per cysts) = spermatids E1 until E3. Together with laser DIC (differential interference contrast), it is thus possible to get an overview over testis morphology. Size and shape of nuclei, as well as their position inside or outside the seminiferous tubules allow for determination of cell type. Outside of the seminiferous tubules, in the interstitial compartment, Leydig cells can be identified by their uniform and weakly stained nuclei of 5 μm in diameter and their appearance in groups. Grids were put over the images and the tubules area and each square of the grid was measured in the ImageJ2 environment [46]. Thus, a ratio of tubules area to interstitium could be calculated for each testis. Immunofluorescence with antibodies anti-Vasa and anti-Pcna was described in detail previously by Thönnes et al. [28]. Vasa is an established and specific germ line cell marker with strongest expression in type A spermatogonia and decreasing signal intensity with further differentiation of germ line cells until entry into meiosis. It was used to facilitate identification of germ line cells. Staining of cells during S-phase of the cell cycle using anti-Pcna antibody served to detect mitotically active germ line cells and to show synchronously developing germ cells inside of a germ line cyst.

## Results

### Selecting dominant and subordinate fish for RNAseq

Eight fish from three families were chosen for RNA sequencing (Table 4). Fish in family 1 were nine months of age, in family 2 they were 12.5 months of age and animals in family 3 were 34 months of age. In families 2 and 3 a single male had been the uncontroversial d-male for five months and five weeks respectively. In family 1, three animals were displaying pale coloration and were frequently engaged in fights with each other. This is typical for fish of this age and size. Only one fish however was observed courting a female and mating. This male was therefore chosen as the dominant fish from this family. While dominant fish did not necessarily have the highest body weight, they displayed higher gonad weights than the subordinate males. Consequently, they also achieved higher GSI values. Furthermore, d-males had heavier (= larger) pituitaries than s-males. In the groups chosen for RNA-Seq, d-males had 11-KT plasma values above 4 ng/ml and s-males ranged from 0.7 ng/ml to 1.7 ng/ml (Table 4).

**Table 4:** Measurements of experimental animals.

family	age	fish ID	length (cm)	body weight(g)	gonad weight (g)	GSI	pituitary (mg)
1	9 months	7S	24	260	0.3	0.12	2.3
		9S	22	210	0.19	0.09	1.3
		8D	23.5	191	0.7	0.37	3.0
2	12.5 months	17S	24.5	265	0.2	0.08	2.5
		18D	24.5	236	0.9	0.38	7.7
3	34 months	30S	38	955	1.25	0.13	6.2
		31S	36.5	929	1.77	0.19	5.1
		32D	40	1149	7.53	0.66	7.9
4	30 months	149S	35	730	0.73	0.1	n.d. (Additional fish for RT-qPCR validation)
4	30 months	158D	34.5	544	1.71	0.33	
5	27 months	170D	30	447	2.16	0.48	
6	39 months	175S	35	599	0.77	0.13	

### Gonad morphology and hormone levels of dominant and subordinate tilapia

**Fig 1:**
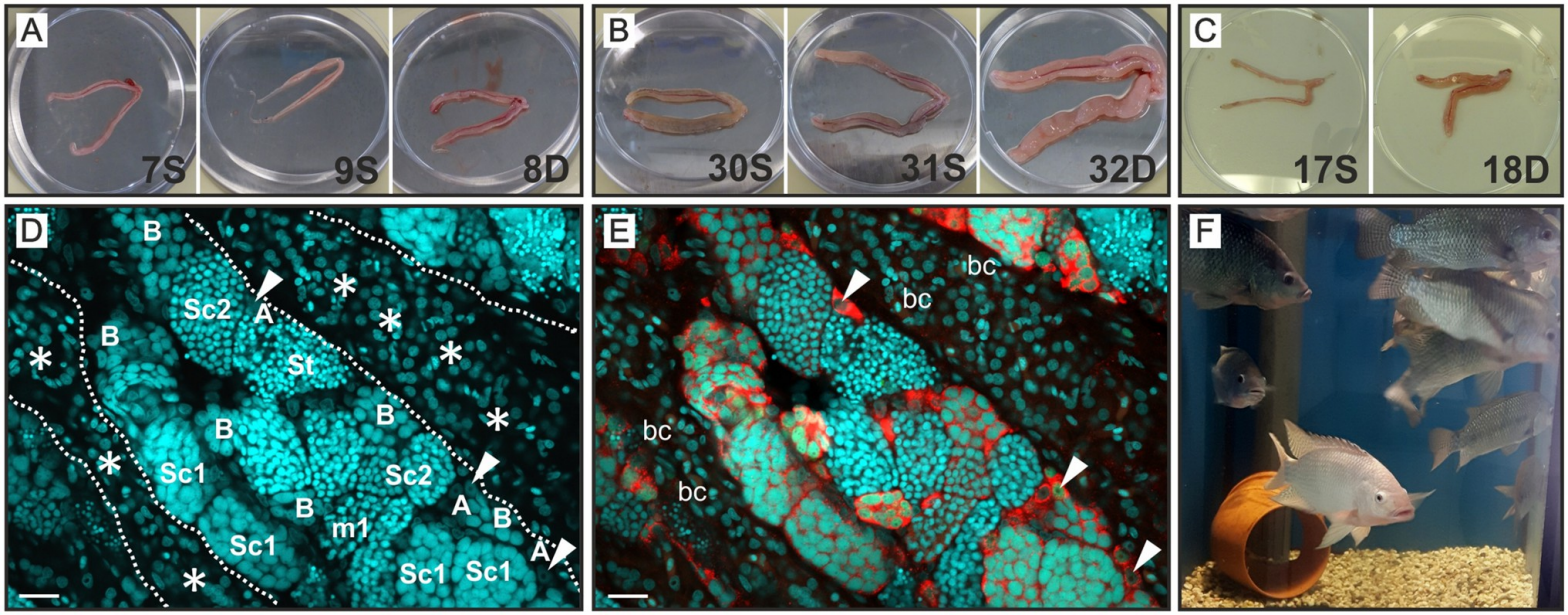
Pictures of testes used for RNA-Seq in this study as well as example images for the testis morphology and a fish tank with Nile tilapia in stable hierarchy. **(A)**, **(B)** and **(C)** Pictures of testes from subordinate (S) and dominant (D) males used in this study (according to Table 4). **(A)** Pictures of family 1 (males IDs 7S, 9S and 8D). **(B)** Pictures of family 3 (males IDs 30S, 31S and 32D). **(C)** Pictures of family 2 (males IDs 17S and 18D). **(D)**, **(E)** Details of testis morphology from a dominant animal (33-month-old male, GSI 0,26; not part of the RNA-Seq experiment) to illustrate morphology of a well-developed testis. **(D)** Draq5^®^ staining (cyan) of cell nuclei. A dotted white line labels position of the wall of seminiferous tubules. Asterisks label interstitial compartment including many Leydig cells (show typical cell nuclei with 5μm in diameter). Germ line cells inside of a tubule were labeled according to their developmental stage: A—type A spermatogonia, B—type B spermatogonia, Sc1 –primary spermatocytes, m1 –Sc1 during first meiotic division, Sc2 –secondary spermatocytes, St—spermatids. A white triangle marks the nucleus of selected type A spermatogonia additionally and these triangles were also inserted in (E). The classification of germ line is based on morphological criteria such as cell nucleus diameter and number of cells per cyst. Single cells with largest nuclear diameters of 8 μm or above inside of seminiferous tubules correspond to type A spermatogonia. Further advanced stages (type B spermatogonia and spermatocytes) have a smaller nuclear diameter then type A spermatogonia and show an increasing number of cells with ongoing generations inside of one synchronously developing germ cell cyst. Spermatids can be easily identified by their high number per cyst and their small nuclear diameter. Further details about staging of germ cells and histological methods are described in the Methods section. **(E)** In addition to the nuclear Draq5^®^ staining (cyan), Vasa protein caused red fluorescence and Pcna protein caused green fluorescence (most of the green fluorescent cells appear in turquoise because of the overlay with a strong Draq5^®^ signal) is shown. The undifferentiated type A spermatogonia have the highest levels of the germ line marker Vasa which is reduced during the further differentiation of germ line cells until onset of meiosis. Pcna is expressed in cells at S-phase of the cell cycle. Blood cells (bc) can be identified in the red/green-fluorescence-channel overlay by their typical brownish appearance within the interstitial compartment. **(F)** Snapshot from a 540 L tank with a long-term stable hierarchy. A dominant white-appearing male holds a territory around the orange tube and at the bottom of the tank. Darker appearing subordinates and females stay outside of its territory. Scale bars in (D) and (E): 20 μm. Diameter of Petri dishes shown in (A)–(C): 90 mm.

All fish used in this study showed normally developed testis morphology. Staining nuclei with Draq5^®^ permitted the identification of different cell types through their nuclear morphology. TA, tubular compartments filled with spermatogonia and spermatocytes and interstitial area containing Leydig cells, blood cells and myoid cells could thus be addressed (Fig 1). Average thickness of TA was 29 μm and 33 μm for s-males 7S and 9S and 69 μm for d-male 8D from family 1. In family 2, TA measured 20 μm for s-male 17S and 110 μm for d-male 18D. In family 3, s-male 30S, which had been displaced as dominant male five weeks before the experiment, had the thickest TA (137 μm), followed by d-male 32D (72 μm) and s-male 31S (69 μm).

In addition to measuring TA thickness, we also evaluated the areas of Leydig cells (interstitium) in relation to the areas of seminiferous tubules in the peripheral testis region (S1 Fig).

The percentage of testis sections filled with Leydig cells tended to be higher in d-males (38%) than in s-males (21%), as shown in S1 Fig. The standard deviation however was relatively large (~26%) for both groups. Comparing the data at the family level revealed that the difference in Leydig cell area between d-males and s-males was not clearly defined. In family 2 the dominant male had more Leydig cells area than the subordinate male. In families 1 and 3, one of the two subordinates was very similar to their respective dominant male. A linear mixed-effects model confirmed that status was not a robust predictor of Leydig cell area in this experiment. The model’s estimate gave a 15% higher Leydig cell area for dominant males, which was however not statistically significant. In this study, we evaluated images taken at the peripheral region of the testes to include the TA. However, large areas of Leydig cells can typically be found near the efferent ducts. The results obtained for the Leydig cell areas in d-males and s-males therefore is valid only for the peripheral, tubules containing regions.

**Fig 2:**
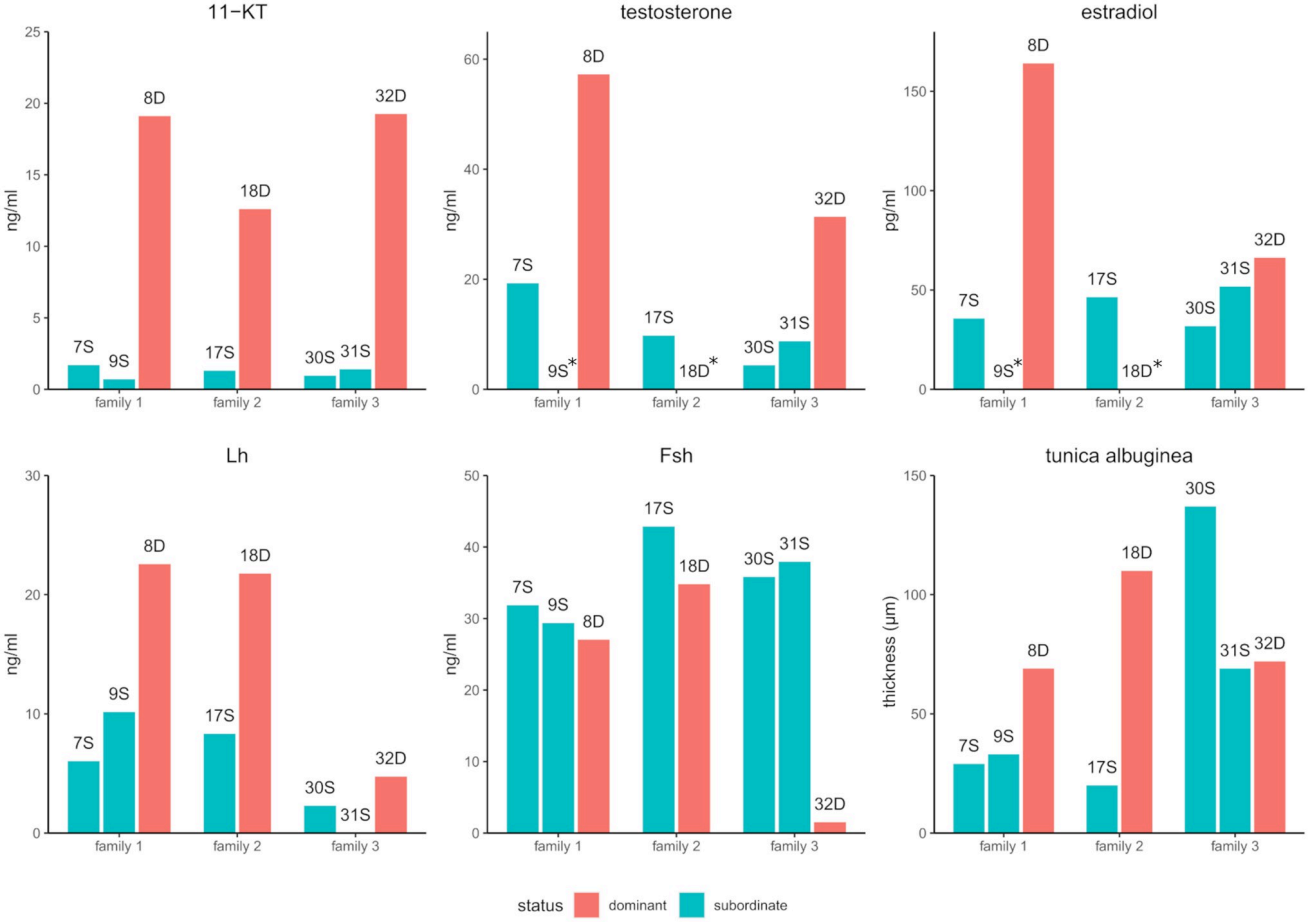
Plasma hormone values and TA diameter. Plasma values of 11-KT, T, E2, Lh and Fsh for each of the experimental animals. Data is represented as obtained by ELISA. Diameter of the TA is also provided for each animal as measured under the confocal microscope. Asteriks (*) marks samples for which hormone measurement was not possible due to limited plasma volume.

Leydig cells produce steroid hormones and thus their presence and number can be used as proxies for steroidogenic capacity. In order to investigate the hormonal status of d-male and s-males more in depth, additional sex hormone levels were measured (Fig 2). Testosterone (T) and 17β-estradiol (E_2_) plasma values were consistently higher in dominant males than in subordinate males. T levels reached from 4–20 ng/ml in subordinate males and lay between 31–57 ng/ml for dominant fish. Although a fixed ratio of T to 11-KT could not be detected, ranking T and 11-KT values yielded very similar results for each fish. E2 levels ranged from 36 to 52 pg/ml in s-males and the two d-males had levels of 66 and 164 pg/ml respectively. Lh values were also elevated in d-males (18–21 ng/ml) when compared to s-males (4–11 ng/ml). Fsh plasma levels were less conclusive and no trend could be inferred from the eight fish tested. The average Fsh levels from two test runs were in the range of 14 to 30 ng/ml. The dominant male from family 3 (ID32) was the only notable outlier. Its average Fsh plasma value was only 4 ng/ml. Both s-males from this family had at least double the plasma Fsh values in both measurements.

### RNA-Seq results reveal differences in pituitary and testis gene expression of d- and s-males

**Fig 3:**
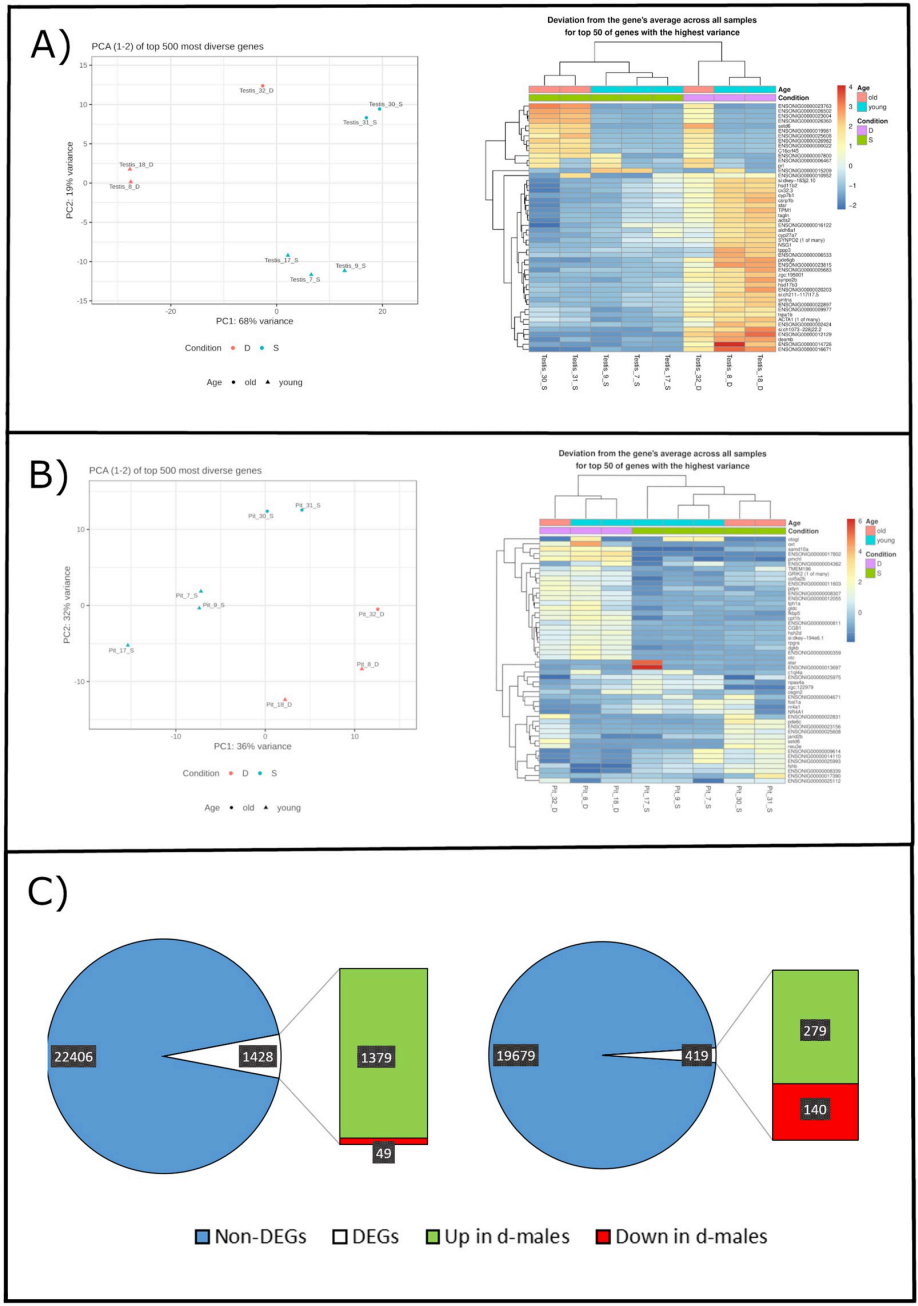
Gene expression profiles of testes and pituitaries from dominant and subordinate Nile tilapia show differences between tissues and social groups. (**A**) and (**B**): PCA plots for DEGs in testes (A) and pituitaries (B) show a clear line of division between dominants and subordinate fish. PCAs were computed based on normalized and log-transformed counts of the top 500 variable genes. A second line could be drawn between family 3 (34 months) and the families 1 and 2 (nine to twelve months). Heatmap clustering for the 50 genes with the highest variance in testes (A) and pituitaries (B) based on normalized and log-transformed counts. (**C**): Numbers of genes sequenced in testes and pituitaries and DEGs used for functional annotation. DEG were defined when the fold change between s-males and d-males had an adjusted p-value of <0.05.

In total, RNA-Sequencing yielded expression data for 23785 genes from testis and 19958 genes from the pituitary gland (S2 Table). 19649 genes were expressed in both tissues. The reads produced by RNA-Sequencing were aligned to the reference genome. For both testis and pituitary, the general alignment rate was above 97%, most of them mapping to only a single position on the reference genome. Half of the reads could be assigned to exonic regions. Table 5 summarizes the alignment statistics. PCA and heatmap analysis revealed clear differences between dominant and subordinate samples (Fig 3).

**Table 5:** RNA-Seq results and alignment statistics.

Tissue	ID	Total Reads			Exonic Region			Fragments uniquely assigned to exonic region	
		Read Count	Aligned	Rate	Proportion of Reads	Transcripts	Genes	Read Count	%
Testis	7S	31808125	31291692	0.984	0.573	21810	12552	19806600	59.6
	9S	32920243	32255298	0.980	0.575	21900	12596	19904791	60.3
	8D	33188668	32445383	0.978	0.58	21816	12583	20165406	60.3
	17S	28232828	27785444	0.984	0.64	22033	12637	21251409	67.2
	18D	28103753	27445079	0.977	0.621	22052	12651	21479140	65.0
	30S	34897413	34107863	0.977	0.552	22277	12740	19649255	57.7
	31S	30665940	30118149	0.982	0.549	22129	12629	20021511	57.6
	32D	32699285	31981819	0.978	0.586	22177	12699	20415845	61.1
Pituitary	7S	33192674	32519008	0.980	0.539	27169	13997	18751102	58.8
	9S	32970343	32340253	0.981	0.496	26807	13851	17636609	53.5
	8D	33431394	32704209	0.978	0.545	26700	13914	19556176	58.8
	17S	31573936	31032120	0.983	0.56	27027	14031	17333599	61.2
	18D	32978992	32333716	0.980	0.54	26470	13883	16391728	58.2
	30S	34031739	33170727	0.975	0.443	27858	14090	16449253	47.1
	31S	34736707	33921134	0.977	0.491	26393	13732	16314332	53.1
	32D	33355749	32542595	0.976	0.506	26933	13910	17871863	54.6

Data analysis revealed that 1606 (testis) and 463 (pituitary) genes were differentially regulated between subordinate and dominant animals (Fig 3). Notably, most up-regulated genes were found in the tissue from dominant males.

### Dominance in male tilapia is reflected in up-regulation of steroidogenesis related genes, growth factors and further BPG-axis related genes

A number of genes involved in the synthesis of steroid hormones were not only strongly expressed in general but also clearly upregulated in d-male testis samples. They include *stAR2* (6243 TPM) which encodes steroidogenesis acute regulatory protein, which is essential for the translocation of cortisol from the cytoplasm to the inner mitochondria membrane [26, 47]. Several hydroxysteroid dehydrogenases like *hsd17b3* (18 TPM), *hsd3b1* (1059 TPM), and *hsd11b2* (1454 TPM) as well as cytochrome P450 family genes like *cyp11a2* (1558 TPM), *cyp11c1* (176 TPM) or *cyp17a2* (1152 TPM) were also upregulated.

Furthermore, receptors related to spermatogenic processes like androgen receptors (*ar*), prostaglandin receptors (*ptger*) or retinoic acid receptors (*rar*) and were also upregulated in dominant males, as were a number of Igf binding proteins (*igfbp*) and components involved in or related to the Wnt-signalling pathway with their expression ranging between 13 and 65 TPM. All essential components for Wnt-signaling were detected in the testis transcriptome and the ones upregulated in the dominant fish comprise a Wnt related dickkopf gene (*dkk3b*), or the Wnt/β-catenin target gremlin2 (*grem2a*). A frizzled gene family receptor (*fzd*), secreted frizzled related proteins (*sfrp*), transcription factor 23 (*tcf23*), transcriptional regulator *myca*, two fibronectin 1 isoforms (*fn1*), two protocadherins (*pcdh*), cadherin 16 (*cdh16*), cadherin like and PC-esterase domain containing 1 (*cped1*) and the transcription factor gene *snai2* were also upregulated. These components of the Wnt-signalling pathway were expressed in the low (3 TPM: *cdh16*, *pcdh18a*, *sfrp5*,) to moderate (278 TPM: *grem2a*) range.

Further relevant pathways in reproduction are controlled via transforming growth factor beta (Tgf-β) signaling. In this context two activin receptors (*acvr2bb*, 12 TPM and *acvrl1*, 31 TPM) were upregulated in d-male testis. Further, two androgen receptors (*ar*, 22 TPM and *ara*, 19 TPM) are also upregulated in d-males. A number of upregulated DEGs are related to estrogen signaling, for example, an estrogen receptor gene (*esr1*, 65 TPM) or *greb1* like retinoic acid receptor coactivator (*greb1l*, 47 TPM). Increased expression was also detected for retinoic acid receptor β (*rarb*, 2 TPM) and the aldehyde dehydrogenase family protein crucial for retinoic acid synthesis (*aldh1a2*, 8 TPM). Another upregulated receptor was Gndf family receptor α (*gfra1*, 7 TPM), typically found in undifferentiated spermatogonia [48] and regulated by Fsh [49]. Four more receptors involved in reproduction were found to be upregulated in d-males: a prolactin receptor (*prlra*, *9 TPM*), two prostaglandin receptors (*ptger1b*, 26 TPM and *ptger4b*, 4 TPM) and a somatostatin receptor (*sstr2a*, 11 TPM). The growth factor *insl3* (1537 TPM) was also upregulated in this context.

**Fig 4:**
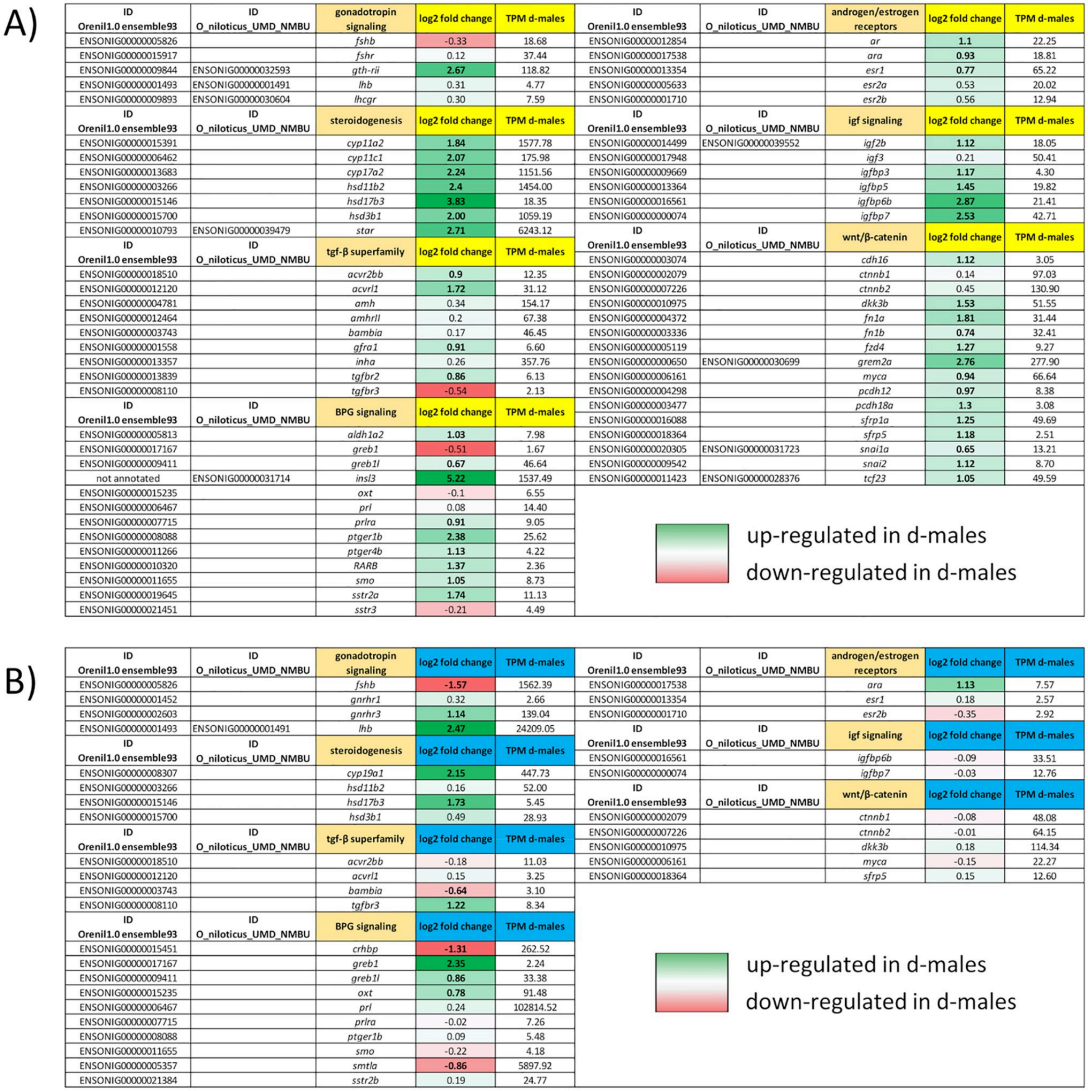
Gene expression in testes and pituitaries of dominant and subordinate Nile tilapia. Expressions of selected genes as measured by RNA-Seq for testis **(A)** and pituitary **(B)** with log2fold change and TPM (transcripts per million). Bold face indicates significant regulation (DEG) with adjusted p-value (pjad) < 0.05. In case the original (Orenil1.0ensembl93) Ensembl gene IDs have retired in the current annotation (O_niloticus_UMD_NMBU), both IDs are listed.

Genes upregulated in the pituitary of dominant males include the brain aromatase gene (*cyp19a1*, 445 TPM), *hsd17b3* (5 TPM) and *gnrhr3* (139 TPM), as well as *ara* (8 TPM), *greb1l* (33 TPM), *tgfbr3* (8 TPM), and oxytocin/isotocin (*oxt*, 91 TPM). For *cyp19a1*, we followed the nomenclature of ensembl.org. The Nile tilapia gene *cyp19a1* (ENSONIG00000008307 on LG7) fulfills the function of a brain aromatase (*cyp19a1b* in zebrafish). Similar to the situation in the testis, most DEGs found in the pituitary were up-regulated in d-males. However, in this tissue about one third of DEGs were downregulated compared to subordinate males. Amongst them was corticotropin-releasing factor-binding protein (*crhbp*, 263 TPM), which may be involved in stress response. Furthermore, somatolactin alpha (*smtla*, 5898 TPM) was downregulated in d-male pituitaries. Contrarily to *lhb* (24209 TPM), follicle stimulating hormone subunit beta (*fshb*, 1562 TPM) was less strongly expressed in the pituitaries of dominant tilapias. Finally, the *bambia* (BMP and activin membrane bound inhibitor (3 TPM) transcript was downregulated as well. A selection of relevant DEGs, their expression in TPM (transcripts per million) and their regulation between social ranks is displayed in Fig 4.

### Pathway enrichment analysis shows activated metabolic activity in d-males

**Fig 5:**
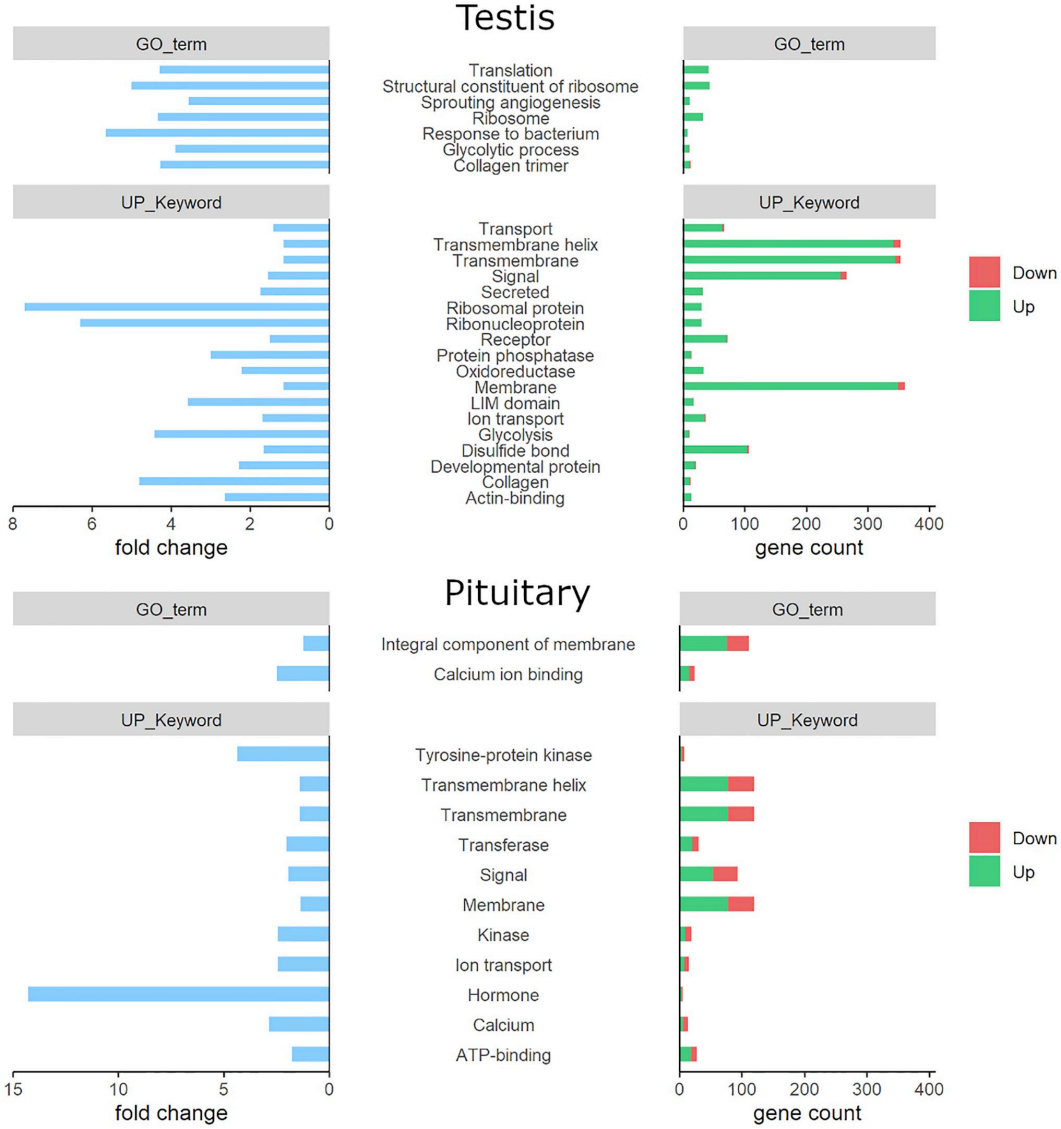
Pathway enrichment with DAVID. Pathway enrichment analysis results displayed as gene count and fold change. All pathways shown here are significantly enriched in the DEG dataset (Bonferroni corrected p-value <0.05).

DEGs from the testes of d- and s-males were examined for enriched pathways with the DAVID tool. In testis, the analysis revealed 18 significantly enriched UP Keywords for the DEG list (Fig 5). Genes belonging to these categories make up a higher percentage in the DEG set than in the whole genome. The most enriched categories are **Ribosomal protein** (8-fold) and **Ribonucleoprotein** (6-fold). Other metabolically relevant categories comprise **Glycolysis** (4-fold) and **Oxidoreductase** (2-fold). UP Keywords grouping terms involved in signaling were also enriched, namely **Signal**, **Secreted** and **Receptor** (2-fold) as well as **Protein phosphatase** (3-fold), **Transport** (1-fold) and **Ion transport** (2-fold) or **Transmembrane** (1-fold) and **Transmembrane helix** (1-fold). Enriched terms related to structure are **Collagen** (5-fold), **LIM domain** (4-fold), **Actin binding** (3-fold), **Disulfide Bond** (2-fold) and **Membrane** (1-fold). **Developmental protein** is also enriched two-fold.

Using GO term annotation, the enriched terms are similar to the ones retrieved from UP Keywords. Especially, **Structural Constituent of ribosome** (5-fold), **Ribosome** and **Translation** (both 4-fold) as well as **Glycolytic process** (4-fold) point to increased protein synthesis and energy conversion in d-males. Furthermore, genes with the tags **Sprouting angiogenesis** (4-fold), **Response to bacterium** (6-fold) and **Collagen trimmers** (4-fold) are enriched in the DEG dataset. Reflecting the findings from the DEG analysis, most genes in the enriched categories were upregulated in d-males (Fig 5). In summary, pathway analysis showed that translation and signaling are significantly activated in dominant male tilapia in comparison to their subordinate counterparts.

In the pituitary, enriched pathways include a number of membrane related terms like the UP-keywords **Transmembrane helix** (1-fold), **Transmembrane** (1-fold) and **Membrane** twofold) or the GO term **Integral component of membrane** (1-fold). Also enriched were Up-keywords **Calcium** (3-fold), **Ion transport** (twofold) and GO term **Calcium ion binding** (twofold). **ATP-binding** (twofold) was another enriched Up-keyword. In contrast to the testis, signaling related term were notably enriched in the tilapia pituitary gene expression. These Up-keywords comprise **Hormone** (14-fold), **Kinase** (twofold), **Tyrosine-protein kinase** (4-fold), **Transferase** (twofold) and **Signal** (twofold). Like in the testis, most genes in the categories named above were up-regulated in d-males. The complete list of functional annotation results can be found in S3 Table.

### Validation of RNA-Seq results by RT-qPCR

The expression of 15 selected genes was tested in testis samples by RT-qPCR, eight of them were DEG according to RNA-Seq. In addition to the eight animals used for the original experiment, two dominant and two subordinates were added to the test set (Table 4). This measure was taken to achieve statistically robust data. Expression was calculated as described in the method section. ANOVA was performed on log2 transformed, normalized data (S4 Table).

**Fig 6:**
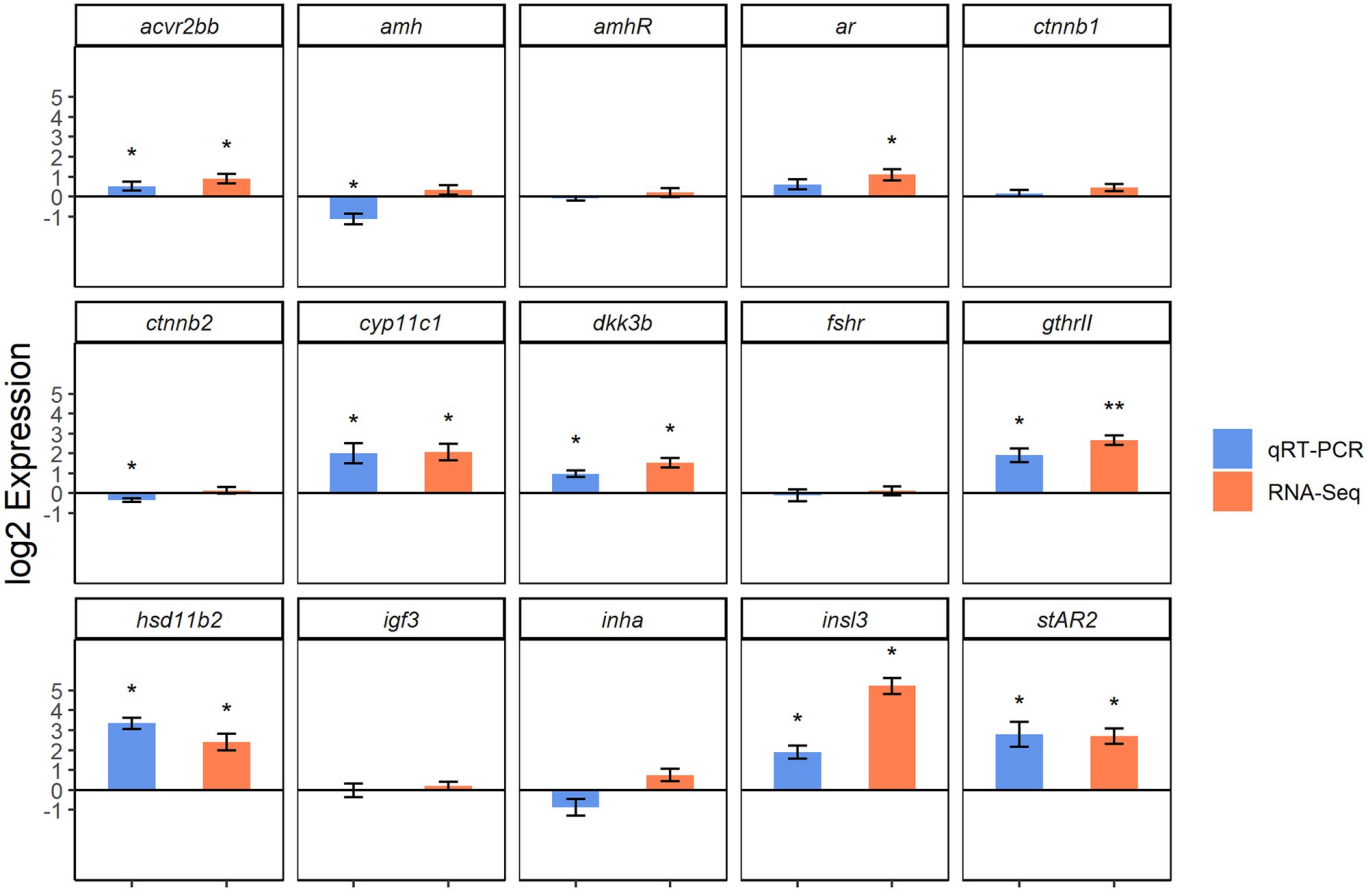
RT-qPCR expression of chosen genes from RNA-Seq. Testis expression of 15 genes measured with RNA-Seq and RT-qPCR of dominant fish compared to subordinate fish. Values are expressed as log2fold change +/- SEM. Significant differences for RT-qPCR data was determined via ANOVA. * = p< 0.05.

For twelve out of 15 genes both RNA-Seq and RT-qPCR experiments yielded the same results (compare Fig 6). *Acvr2bb*, *insl3*, *cyp11b2*, *dkk3b*, *gthrII*, *hsd11b2* and *stAR2* were upregulated DEGs in d-males and *amhRII*, *inha*, *ctnnb1*, *fshr* and *igf3* were not affected by fish status neither in the transcriptome analysis nor in the qPCR validation experiment. In contrast, *ctnnb2* and *amh* were not expressed differentially in the RNA-Seq experiment, RT-qPCR however showed a downregulation of these two genes. The gene encoding the androgen receptor *ara* on the other hand was upregulated in the RNA-Sequencing but not affected by status in the RT-qPCR.

## Discussion

### Hormone values reflect the differences found in the transcriptomes

In addition to histological inspection, steroid hormone levels were measured in this study to evaluate the actual steroidogenic levels of the experimental animals. 11-KT was used as a diagnostic value to identify suitable animals for RNA-Seq (Table 4). Steroid ELISAs on plasma from those fish revealed that Testosterone (T) and Estradiol (E2) were found at higher concentrations in d-males compared to s-males (Fig 2). Higher steroid levels in dominant fish can result from either a higher number of Leydig cells, increased gene expression of steroidogenic enzymes or a combination of both.

DNA staining with Draq5^®^ facilitated identification of cell composition in vibratome sections under the confocal laser scanning microscope (compare Fig 1). Leydig cells and germline cell filled tubules could be identified this way. In our study, dominant males tended to have more Leydig cells than subordinate males, even though the difference was not statistically significant. The higher GSI of d-males in comparison to s-males is another point of evidence for a higher number of Leydig cells in the former, as the two factors are positively correlated in general [50]. Considering the fishes’ ability to quickly ascend in rank when a possibility presents, it makes sense that also s-males retain a number of Leydig cells. Thus, they can ramp up steroidogenesis and sperm production quickly [18]. When examining complete testis cross section, d-males indeed have a higher percentage of Leydig cells compared to s-males [13]. In the present study however, Leydig cell areas were not evaluated across the entire cross section. In order to measure thickness of the TA, sections near the periphery of the testes were chosen for evaluation. This is also where most of the blind ending tubules are located. Consequently, interstitial tissue made up a larger proportion of testis tissue towards the efferent ducts. Due to the fact that vibratome sections from s-males were smaller in means of area compared to those of d-males, the Leydig cell containing interstitium was over-represented in analyzed sections from s-males. Therefore, we could not identify the final causation (elevated expression of steroidogenesis genes, higher number of Leydig cells or a combination of both) of this finding, but in this study, we saw clearly elevated plasma 11-KT levels in d-males.

The interrelation of sex hormone levels, sex hormone receptors and social status have been well documented in the literature [50–52] and were also evident in the present study. The main place of sex steroid production in males are the Leydig cells in the testis, however, steroid production and conversion are also known to happen in the brains and pituitaries of teleost fish [7, 53, 54]. Brain aromatase (the gene is referred to in this work as *cyp19a1*), converts androgen into estrogens. In a whole brain gene expression study on *Astatotilapia burtoni*, Renn *et al*. [55] found that the brain aromatase gene was higher expressed in dominant males. Similar results we obtained in the present work, even though here the expression data come exclusively from the pituitary gland. Our RNA-Seq of *O*. *niloticus* pituitaries revealed *cyp19a1* significantly higher expressed in dominant males. Another study from Huffman *et al*. [56] showed that inhibition of brain aromatase gene in the POA of the brain of *A*. *burtoni* reduced aggressive but not reproductive behavior. All these findings could be interpreted as estrogens indirectly promoting reproduction by aiding the maintenance of dominance in social cichlids. Further studies, which focused at the pituitary gland, identified *brain aromatase* to be specifically expressed in Lh cells of the pituitary of some fish [8], suggesting a direct effect of sex steroids on Lh cells activity. Based on the literature discussed above and our own findings, we assume that estrogens are involved in the development of social structures by influencing the brains and pituitaries of fish.

Androgens on the other hand also have an undoubted importance for male reproduction. Consequently, we identified a number of DEGs involved in androgen synthesis and androgen signaling in the present study as shown in Fig 4. Some, like the gene encoding the enzyme catalyzing the conversion of androstenedione to testosterone (*hsd17b3*) or one of the androgen receptor genes (*ara*) were upregulated in both testes and pituitaries of d-males. Both genes showed moderate expression levels (5–19 TPM) but significant differences between dominant and subordinate males. Other genes were upregulated only in the testes of dominant males. Among these were for example the highly expressed (TPM > 1000 in d-males) genes *stAR*, *cyp11a2*, *cyp17a2*, *hsd11b2*, *hsd3b1*, as well as *cyp11c1 (176 TPM)*, androgen receptor *ar* (22 TPM) and an estrogen receptor *esr1 (65 TPM)*. Cortisol is converted to pregnenolone by Cyp11a2 before Hsd3b1 processes it to progesterone. Cyp17a2 is then involved in converting progesterone into androstenedione. Androstenedione in turn gets converted into testosterone and 11-ketotestosterone by Hsd11b2, Hsd17b3 and the Cyp11 enzymes. For a current review of the steroidogenic pathways in teleosts see Tenugu *et al*. [57] or Rajakumar and Senthilkumaran [9].

These findings fit the fact that d-males had higher plasma levels of E2, T and 11-KT and also show that sensitivity to androgens could be increased due to the overexpression of the respective receptors.

### Lh appears to be an important factor in supporting upregulation of genes related to long-term dominance

All vertebrates rely on gonadotropin releasing hormones (Gnrh) to initiate the release of gonadotropins (Gths) from the pituitary gland. Their crucial role in reproduction and dominance have been studied in the past [58–61]. Gth receptors therefore constitute a gateway in the Gth signaling chain. Looking at differential expression of *gnrhr* is therefore the logical next step of investigating how social status influences gene expression along the BPG-axis. In the present study, we identified two *gnrhr* genes (ENSONIG00000002603 = *gnrhr3*, 139.0 TPM, and ENSONIG00000001452 = *gnrhr1*, 2.7 TPM) which were expressed at meaningful levels in the pituitaries and *gnrhr3* was expressed at significantly higher levels in d-males than in s-males. This finding agrees with the literature where Gnrh has been linked to stable social dominance in *A*. *burtoni* [55, 62, 63], medaka [64] and Nile tilapia [65]. The study by Ogawa and Parhar (2020) furthermore showed that *gnrhr1* (referred to as *gnrhr3* in our study) expressing neurons of dominant tilapia males also express more estrogen receptor subtypes than those found in subordinate fish. This finding again points to an involvement of estrogens in social dominance. A recent study by Hollander-Cohen *et al*. [66] further showed that Gnrhr3 is enriched in Lh-cells from tilapia pituitaries while Gnrhr1 is enriched in Fsh-cells.

Measuring plasma levels of the two gonadotropins revealed that in the present study Lh levels were higher in dominant fish than in subordinate ones. This was corroborated by the massive expression of *lhb* in the pituitaries of dominant males. With 24209 TPM it was among the top ten genes with the highest expression in the pituitary of d-males, a number 6.8 (log2 fold change = 2.47) times higher than in s-males. The gene encoding the beta subunit of Fsh (*fshb*), the other gonadotrope hormone, on the other hand was downregulated in the pituitaries of dominant males (1562 TPM) when compared to subordinate tilapia (6227 TPM). Concerning plasma levels of Lh, we could not find a significant difference between the social groups, however. Contrasting the differences of gonadotrope gene expression at the pituitary level, the receptors *lhgcr* and *fshr* did not reflect them at the testis level. While conserved throughout vertebrate taxa, Lh and Fsh function differently in teleosts than in mammals. This is exemplified by the fact that in the teleost pituitary there are two types of gonadotropes, secreting either Lh or Fsh. Their mammalian counterparts on the other hand can produce both hormones in the same cells. Regarding their target cells the situation is somehow reversed. Receptors for FSH and LH are highly specific in mammals and are only expressed on certain cells of the testis (FSH—Leydig cell, LH—Sertoli cell) [1, 5, 67]. In many fish, Sertoli and Leydig cells express both receptors [1, 68–70]. In addition, both hormones and receptors appear to be at least partly redundant in zebrafish [71–73]. On the other hand, evidence has begun to emerge that in other teleosts like *O*. *niloticus* Lh and Fsh serve distinctive but as of yet not fully elucidated purposes [74]. The present study would add further evidence to this case. For example, one could hypothesize based on our present findings that Fsh is crucial in activating reproductive pathways but their maintenance in long-term d-males relies on other hormones, like Lh.

### Transcriptome analysis revealed clear differences between s- and d-males both in testis and in pituitary

#### Social status and age cause differently expressed genes

Heatmap clustering and Principal Component Analysis (PCA) showed that social status explains differences in gene expression between the two groups very well (Fig 3). This is consistent with previous studies investigating the gene expression in the brain of dominant and subordinate fish [14, 50, 75]. As we expected, samples grouped most clearly by social status. However, a second line of division could be drawn between family 3 (30S, 31S and 32D) and the other fish (Fig 3). The most striking difference between those families was the age of the fish (nine and twelve months vs. 34 months). Ageing and senescence are not very well understood processes yet, neither in mammals and even less in fish [76, 77]. Early development from egg to embryo to larva are well-defined stages of the fish lifecycle. The transition from adolescent to adult can also be pinpointed to the reaching of sexual maturity. When an animal has passed the prime of its life and senescence starts is much harder to define however, especially as many animals are able to produce offspring during the whole duration of their lifetime. According to fao.org [78], Nile tilapia can reach over ten years of age. With a little short of three years the fish in family 3 would have just reached a third of that timespan. Tilapia are valued for their fast growth which means they reach slaughter weight quite early under the right conditions. Morais *et al*. [79] report weights of over 1000 g for eight months old Nile tilapia, for example. To the authors’ knowledge, tilapia used in scientific contexts are also usually not older than 12 months. Therefore, our results show that the study of tilapia of more advanced age could still provide new insights into their biology. Especially the effect of aging on the BPG-axis and therefore on fertility could be promising.

In the testis, the enrichment analysis with DAVID yielded a number of translation and ribosome related terms as well as terms associated with signal transduction and general metabolism. In the pituitary the situation was similar. However, in this tissue we saw a narrower range of enriched terms. In the pituitary, most significantly enriched categories could be linked to hormone production and regulation as well as signaling in general. Most studies focusing on gene expression in social phenomena like dominance have looked at the brain as a whole [55, 56, 75, 80]. This makes sense, as conserved structures involved in dominance behavior are located in the brain, like the Social Decision Making Network (SDMN) and specifically the POA (preoptic area) [50, 81–83]. The intricate signaling networks in teleost pituitaries are just beginning to be described [2, 3, 67, 84, 85]. Thus, transcriptome studies like ours provide important data for unraveling yet unknown pathways in the pituitary which are involved in dominance.

#### Social dominance is reflected on the transcriptional level by upregulation of genes related to spermatogenesis and gonadotropic stimulation

*The contribution of growth factors like Insl3*, *Igf*, *prolactin and somatolactin to the implementation of social dominance*. One of the most strongly affected DEGs in the testes of d-males and s-males in the present study was relaxin family growth factor *insl3*. This gene has been identified in a numerous vertebrates, including teleosts [86, 87]. In male mammals as well as in fish, *insl3* is expressed exclusively in Leydig cells [88, 89]. To date, the best insights into the role of *insl3* in teleost spermatogenesis stems from studies in zebrafish. Here, tissue culture and *in vivo* experiments revealed that Insl3 is highly sensitive to Fsh (but not Lh) stimulation and induces proliferation of undifferentiated spermatogonia, independently form androgens [68, 88, 90, 91]. A recent knock-out study in zebrafish further succeeded in starting to unravel the downstream actions of *insl3* [92]. The researchers showed that besides direct stimulation of undifferentiated spermatogonia via the Rfxp2a receptor, Insl3 can utilize several paracrine mechanisms to regulate the spermatogenic process, especially via Sertoli cell actions (retinoic acid and proliferator-activated receptor gamma signaling). In tilapia, *insl3* is far less understood. Thus, the findings of the present study underline the versatile role *insl3* plays in teleost reproduction by linking its expression to increased spermatogenic activity and dominance in Nile tilapia, especially since the gene showed a considerable strength of expression with 1537 TPM in d-male testes. Our results also lead to speculate that further potent stimulators of *insl3* apart from Fsh, for which fish of both ranks had similar levels in this study, exist.

Another important class of genes for growth and reproduction are insulin like growth factors (*igf*). Igf3, a teleost specific growth factor is expressed predominantly in adult gonads [93] and is a highly relevant promoter of spermatogonial differentiation [25, 94, 95]. In zebrafish, *igf3* seems to be expressed mainly in Sertoli cells contacting pre-meiotic spermatogonia [94] while in tilapia it was also localized in differentiated spermatogonia and even spermatids [95]. Androgen insufficiency caused down-regulation of *igf3* in both zebrafish [91] and tilapia [25, 96] and for stimulation this gene relies on gonadotropins via cAMP signaling [25, 94]. In the present study, *igf3* itself was not differentially regulated between the two social ranks, but we found a number of Igf binding proteins (*igfbp*s) amongst the DEGs upregulated in d-males. This means, Igf signaling could be activated through their binding proteins.

Igfbps bind to Igfs and thus modulate their bioavailability and activity [97, 98]. They are found most commonly in the liver but also other tissues like the gonads [99–102]. Expression of *igfbp*s in fish is influenced by growth hormone and thyroid hormone [103–105], estrogenic compounds [105], Fsh, 11-KT, Igfs [106–108] and Lh [108]. Studies in zebrafish have shown that Igfbps regulate Fsh induced spermatogenesis by modulating Igf3 bioactivity and thus balancing differentiation and self-renewal of undifferentiated spermatogonia [106, 107]. In the present study, *igfbp5a*, *igfbp3*, *igfbp6b* and *igfbp7* were upregulated in d-male testis with expression levels between 4 and 43 TPM. According to Wang *et al*. [97] *igfbp5* is involved in anabolic metabolism in teleost muscles. In zebrafish, it was found that *igfbp5b* is expressed at much higher rates than *igfbp5a* and that the latter is not regulated by Fsh nor 11-KT [109]. Experiments with trout revealed that *igfbp6* is regulated by Fsh and to a lesser extend Lh and probably androgens (indirectly shown by use of trilostane) [108]. Safian *et al*. [107] showed that Fsh and Igf3 both decreased the transcription levels of *igfbp1a*, *igfbp3* and *igfbp6a* and Fsh increased transcription of *igfbp2a* and *igfbp5b*. Taken together, Igfbps in the testis are potent modulators of spermatogenesis and may be of special relevance in long-term dominance by protecting fish against depletion of undifferentiated spermatogonia [106]. This would fit our finding that several Igfbp genes were upregulated in the testes of dominant tilapia.

Insulin like growth factor 2 (Igf2) is a regulator of embryonic growth in vertebrates, in fish however, it plays a role in adulthood as well [110]. The number of *igf2* copies in fish appears to be variable. While zebrafish and cave fish possess *igf2a* and *igf2b*, only one copy of the four exon gene was found in the genomes of ten other fish, including tilapia [111]. Although predominantly expressed in the liver, Caelers *et al*. [112] detected *igf2* expression in a number of other tissues in Nile tilapia, including gonads. In our study, testicular *igf2b* was significantly up-regulated in dominant males. From the literature, little is known about the function of gonadal *igf2* and most studies on *igf2* regulation focus on the liver. In primary hepatocyte culture from *O*. *mossambicus* for example, researchers found a stimulating effect of growth hormone, insulin and cortisol on *igf2* transcription. According to the authors, this is typical for fish and resembles the regulation of *igf1* in mammalian livers [113]. In *O*. *mossambicus*, recombinant Igf2 as well as Igf1 were shown to stimulate general growth [114] and both Igfs further inhibited growth hormone and stimulated prolactin secretion in this species [115]. Igf regulation by steroid hormones is well investigated in vertebrates including fish and in general testosterone increases *igf* expression, while estradiol has a contrary effect [116]. In the context of our present results, upregulation of testicular *igf2* is in concordance with elevated sex steroid levels and up-regulation of other growth factors (mentioned above) in dominant males.

The growth hormone family protein prolactin (Prl) and its receptors have been detected in a number of tissues and cells, mainly in the pituitaries [117], but also the testes of teleost fish [118–120]. Prolactin plays a vital role in osmoregulation [120, 121] and immunity [122] but has also been reported to be involved in sexual maturation, spermatogenesis, ovulation and steroidogenesis in different fishes [117]. Although *prl* was a DEG neither in the testes nor the pituitaries in the present study, we found an upregulation in one of the receptors (*prla*) in the testes of d-males. Therefore, one could speculate that steroidogenesis inducing actions of gonadotropins are mediated by prolactin receptor abundance rather than prolactin levels in Nile tilapia testes. Interestingly, a tissue culture experiment by Rubin and Specker [123] with *O*. *mossambicus* testes showed exactly that. Equal prolactin hormone levels can still have different effects, depending on the social context. In their study, Prolactin exposure alone resulted in testosterone production in testes of courting males but not in those of so called “bachelor” males which were housed in male-only groups and were similar in coloration to females. The study also discovered that Prl mediated Lh effects differently in the two groups. In the presence of both hormones, testes from courting males released even higher amounts of testosterone than when treated with Lh alone. In non-reproducing fish however, Prl decreased Lh induced testosterone secretion. Recently, it was further reported that prolactin transcripts were enriched in the Lh secreting gonadotrophs of male Nile tilapia [66]. In summary, our present study adds another clue as for the involvement of prolactin in modulating hormone levels in dominant and subordinate fish.

Somatolactin is another member of the family of growth hormones and specific for fish. Basal taxa like eels, catfish, salmons and carp usually feature two somatolactin genes while one is typically found in more derived species like tilapia [124–126]. Its functions include involvement of chromatophore regulation, metabolism and reproduction [124], for example by stimulating spermatogenesis in coho salmon [127]. In the present study, somatolactin encoding *smtla* was stronger expressed in the pituitaries of subordinate tilapia. This finding contradicts a study by Renn *et al*. [55] on whole brains of dominant and subordinate *A*. *burtoni*, where somatolactin expression was higher in d-males than in s-males. Neurokinin B (NKB) and NKB- related peptide were shown to increase somatolactin production in grass carp pituitaries [128]. This stimulation was even stronger in the presence of Igf1 or Igf2 with the Tac3 receptor as a functional link between both signaling pathways [129, 130]. In the present study, we did not find overexpression of *tac3* or Igf related genes in the pituitaries of the experimental animals. Therefore, one could hypothesize the existence of other ways of transcriptional control of *smtla*, especially in taxa which have lost one of the two somatolactin orthologues present in basal teleosts. How and if *smtla* expression is related to social status in cichlids still needs to be investigated further.

*Expression of Wnt-*, *retinoic acid- and Tgfβ-signaling components in males with different social status*. Many components of the Wnt/β-catenin signaling pathway could be found in the transcriptome data generated in the present study (S2 Table). In the testes of dominant males, a number of related genes were overexpressed (Fig 4). The two β-catenin paralogues *ctnnb1* and *ctnnb2* however were not differentially expressed between the two social ranks. RT-qPCR confirmed this finding in the case of *ctnnb1*, whereas *ctnnb2* was slightly downregulated in dominant males. *Dickkopf3b* (*dkk3b*) on the other hand was up-regulated in testes of d-males in both in the RNA-Seq as well as in the RT-qPCR experiment. Wnt/β-catenin signaling was traditionally seen as a female promoting pathway, today however its activity has been documented in all stages and aspects of testis development as reviewed elsewhere [131]. Nor is Wnt/β-catenin signaling imperatively a repressing force in male development, as has been shown in the case of mice where active Wnt/β-catenin signaling is necessary for spermatogonia differentiation [132, 133]. Studies on zebrafish also concluded that Fsh triggered β-catenin signaling and promotes proliferation of undifferentiated spermatogonia, for example via the non-canonical *wnt5a* or [90, 134] by stimulating *igf3* expression which in turn activates the canonical β-catenin pathway [135]. Mammalian DKK3 and teleost *dkk3a* and *dkk3b* encode glycoproteins of the dickkopf family which however do not bind to the LRP5/6 receptor [136]. Nevertheless, Dkk3 appears to stimulate Sertoli cell maturation in mammals during puberty by suppressing Wnt4/β-catenin signaling [137]. Leonard *et al*. [138] further discovered a second splice variant of *Dkk3* in mice which was shown to suppress Wnt-signaling by scavenging unphosphorylated β-catenin. Studies in zebrafish further suggest that *dkk3b* is involved in juvenile transition from ovaries to testes and is generally more strongly expressed in male gonads [139, 140]. Keeping in mind these examples of “pro-maleness” capacity of Wnt/β-catenin signaling, our findings of up-regulation of Wnt/β-catenin signaling in d-male testes are backed up by the literature and fits its close ties to spermatogenesis.

The genes *aldh1a2*, which synthesizes retinoic acid, and *rarb* (retinoic acid receptor b) were also upregulated in the testes of dominant tilapia males in the present study. Retinoic acid (RA) is a molecule crucial for spermatogonial proliferation in mammals because it stimulates the expression of meiosis inducing *Stra8* (for review see [141–143]). Although fish like zebrafish, medaka or Nile tilapia do not possess the *stra8* gene, RA has similar effects on teleosts as it has on mammals [144–147]. The upregulation of both *aldh1a2* and a retinoic acid receptor in d-males we found in our experiments therefore hints at increased meiotic activity in the germline of dominant males.

Another gene overexpressed in both pituitaries and testes of dominant fish in the present study was *greb1l* (growth regulation by estrogen 1 like). This cofactor of retinoic acid is important for development of the kidneys, hearing and also the gonads in mammals as well as in fish. Recent studies have further managed to establish a connection between migratory behavior, which is dependent on sexual development, and *greb1l* genotypes [148, 149]. The implications *greb1l* expression has for fish reproduction, as well as its regulation however still remain to be elucidated. The fact that in our study the Rarb gene was also overexpressed in the testis of d-males opens a possible way to explain the involvement of its cofactor *greb1l* in spermatogenesis [147, 150, 151]. Greb1 on the other hand is an estrogen responsive paralogue of the aforementioned *greb1l*, a cofactor of estrogen receptor and indispensable for gene expression mediated by the estrogen receptor [152, 153]. This gene is conserved among vertebrates [154] and in teleosts its main location of expression is the pituitary the development of which it is involved in [155]. Knockout of *greb1* in zebrafish caused a decline in pituitary somatotropes, thyrotropes, lactotropes and gonadotropes and consequently reduced secretion of Fsh and Lh [155] while E2 treatment of pituitaries from grass carp increased gonadotropin secretion [156]. These studies show the influential role of *greb1* on gonadotropin release in fish. In the present study, *greb1* was one of the genes overexpressed in the pituitaries of dominant males. A similar up-regulation of *greb1* in high-ranking males has also been observed by Eastman *et al*. [81] in *Gymnotus omarorum*. In social teleosts therefore, we could hypothesize that *greb1* acts upstream of Lh and Fsh and enables elevated gonadotropin levels in dominant fish.

The Tgfβ superfamily comprises a number of genes crucial for gonadal development and function and its members are well conserved in the animal kingdom. The superfamily comprises Tgfβs, inhibins, activins, Bmps, Amh, Gdsf and distantly related Gdnf [157]. In fish, Tgfβ components are probably of special interest when it comes to sex determination [158]. This hypothesis is based on the still growing number of species which utilize members of the Tgfβ family as sex determining genes [158–167]. In several Nile tilapia strains, copies of the *amh* gene has been found to play the role of male sex determining gene [158, 164, 165, 168, 169]. This point will be discussed later in relation to the assessment of *amh* gene expression level. Amh and its type-II receptor were also recently described in the ovaries, brains and pituitaries of female tilapia [23]. In our transcriptome approach we found expression of *amh* and *amhrII* in the testis only. This indicates a sex-specific difference between male and female pituitary gland regarding Amh signaling. In the RT-qPCR validation (discussed below) however, *amh* was significantly downregulated in the testes of d-males, similar to the results of a previous study from our lab [13]. Amh in fish testes has been shown to inhibit spermatogonial proliferation and steroidogenesis [11, 170–173]. A lower level of *amh* transcripts in dominant males could therefore indicate an involvement of this gene in maintaining a high level of spermatogenesis.

Tgfβ-signaling in general involves a type-I receptor binding to a type-II receptor and subsequent phosphorylation of intracellular Smads. In the tissues we examined, four receptors were overexpressed in dominant males. In the testes, these were *acvr2bb*, *tgfbr2* and *acvrl1* and in the pituitary we found elevated mRNA levels for *tgfbr3*. The activin A receptor type 2Bb (*acvr2bb*) gene was up-regulated in the testes of dominant males. Activin, inhibin and myostatin are ligands of Acvr2b, therefore, testes of dominant males could be more susceptible to the actions of these factors. The same line of argumentation applies to the upregulation we found for activin A receptor type I (*acvrl1*) in d-male testes. Acvrl1 (also called Alk1) is a type-I receptor which can bind Tgfβ and Bmps (with Tgfbr2) as reviewed by [174].

Tgfbr2 (type-II receptor) is part of the canonical Tgfβ-signaling pathway and recruits Tgfbr1. Tgfbr3 (also known as betaglycan) on the other hand, lacks kinase activity. It can act as a co-receptor of Tgfbr2 but also has independent functions, most importantly as a tumor suppressor (for recent review see [175]). Bone morphogenetic protein and activin-membrane-bound inhibitor (Bambi) can inhibit Tgfβ signaling by acting as a pseudo-type-I receptor and interacting with a wide range of type II-receptors (for review see [176]). The down-regulation of *bambia* in the pituitaries of dominant males is another example for the involvement of Tgfβ-signaling in social dominance in tilapia.

Another indication that social dominance in male tilapia goes hand in hand with a higher rate spermatogenic stem cell (SSC) maintenance comes from our findings regarding the expression levels of a neurotrophin receptor from the Tgfβ super-family. In the testes *gfra1* was upregulated compared to s-males. This gene encodes a receptors for Gdnf (glial cell-derived neurotrophic factor) which it can mediate independently or as a cofactor of RET tyrosine kinase [177]. *Gfra1* expression in the testis was restricted to undifferentiated spermatogonia in tilapia [48], dogfish [178] and trout [49] and so far only the neotropical jundía appears to fall out of this pattern [179]. Little is known about *gfra1* regulation in teleosts but *in vitro* experiments in trout revealed that Fsh stimulates *gfra1* expression in the testis [49]. Studies on high temperature effects on tilapia further found that loss of undifferentiated spermatogonia coincides with reduced *gfra1* expression [180]. Preservation of SSCs thus appears to be a conserved role of *gdnf*/*gfra1* action in basal vertebrates as well as in mammals (for review see Fayomi *et al*. [181]). Taken together, our transcriptome data provides valuable evidence for the presence of neurotrophin receptors in the adult testes of teleosts. Moreover, we could show *gfra1* expression correlates with social status and is a promising candidate for functional studies in fish.

*Somatostatin-receptors and further components of the BPG-axis in the context of social dominance*. In 1973, Brazeau et al. [182] discovered that somatostatin (Sst) inhibits the release of growth hormone from the pituitary. Since then, a number of somatostatins and somatostatin receptors in various tissues and with different functions have been discovered in a wide variety of animals, including fish [183–187]. The best studied roles of somatostatin are the aforementioned regulation of growth hormone secretion from the pituitary [184] or its role in metabolism via Igfs [116, 188, 189]. In the present study, we did not find differences in the expression of growth hormone genes between d-males and s-males, therefore it is not surprising that the same held true for sstr2a and sstr3 (expressed in the testis) as well as sstr2b (expressed in the pituitary). However, there is also emerging data pointing towards somatostatin signaling in reproduction. A study by Pillon et al. [190] for example showed that somatostatineric neurons are probably receptible to estrogen and thus regulation by sex steroids. A knockout-study in zebrafish revealed that sst4 -/- animals did not only grow faster than wildtype animals, but displayed delayed onset of spermatogenesis at puberty and downregulation of many genes crucial for spermatogenesis like amh, igf3, insl3 and a number of steroidogenesis related genes [191]. The null mutants further showed lower brain levels of lhb and fshb. First insights into somatostatin actions on behavior comes from two studies in A. burtoni. Using Sst antagonists and agonists, Trainor and Hoffmann [192] could increase and decrease aggressive behavior in male A. burtoni, while reproductive behavior remained unchanged. Although the researchers could not find differences in sstr expression between dominant and subordinate males, they discovered a negative correlation between testicular sstr3, aggression and androgen levels. Based on these results, a study on sstr2 and sstr3 in the brains (hypothalamus) of dominant and subordinate A. burtoni suggests that both localized expression and downstream effects are dependent of social status in this social cichlid [193]. In our study, sstr2a expression was significantly higher in d-males than in s-males in the testes. Although this adds to the growing evidence for the involvement of sst signaling in fish reproductive behavior, possibly via regulation of growth factor or steroid hormones, the implications of these differences in gene expression need to be investigated further.

In the present study, two prostaglandin E (PGE) receptors, *ptger1b* and *ptger4b*, were up-regulated in the testes of dominant males. Prostaglandins are eicosanoids (polyunsaturated fatty acids with hormone like functions) and can be found in a variety of tissues in vertebrates. Prostaglandins appear to be involved in female sexual behavior, ovulation [194–196], and have been correlated to Gth secretion in teleosts [197, 198]. As reviewed by Sugimoto *et al*. [199], PGE receptor 4 seems to be involved in a number of processes involving cell migration, for example gastrulation of zebrafish embryos. In human ovarian cells, *ptger1* and *ptger2* were shown to be involved in steroidogenesis and steroid hormone conversion by 11bHSD1 [200]. Prostaglandins and their receptors are well known for their implications in immune response, acting as both pro- and anti-inflammatory modulators at times and especially their interplay with non-steroidal anti-inflammatory drugs (e.g. aspirin) has been a focus point for research in mammalian models [199, 201]. In fish, prostaglandins were first detected in testis and semen in 1973 by Nomura and colleagues [202] and by now PGE2 has been found in a wide variety of fish cells [203]. Data from a recent study in *O*. *niloticus* further suggests that PGE2 levels are sensitive to estrogenic compounds and pituitary extract [204]. Systematic research into the role of PGE and its receptors in male reproduction of fish is still scarce but given the circumstantial evidence provided above, we could speculate that in the context of social dominance, PGE2 receptor expression is stimulated by sex steroids and gonadotropins and in turn modulate steroidogenesis and stress response.

The gene *gth-rII* was upregulated in the testes of d-males in our study. This gene encodes a receptor for thyroid stimulating hormone (TSH) which is produced in the pituitary. The thyroid axis is known to affect many aspects of fish biology including reproduction at all levels of the BPG axis reviewed by Deal and Volkoff [205]. The expression of TSH receptors in fish gonads has been described for several species and a spermatogenesis enhancing/protecting effect has been postulated [205, 206].

The fish homologue of oxytocin (Oxt), often called isotocin, is a nonapeptide produced by neurons of the POA of the brain which innervate the pituitary and is an important modulator of social behavior [207]. In the present study, *oxt* was up-regulated in the pituitaries of dominant males. For the very closely related species *O*. *mossambicus* it was shown that also the pituitary has high levels of oxytocin, even higher than the examined parts of the brain [208]. In the same study, the researches could show a significantly higher oxytocin level in the hindbrains of socially dominant males and highlighted oxytocin as one of the hormones orchestrating the social phenotype. Although the specific effects of oxytocin are different between vertebrate groups, a common and conserved function appears to be the sensitization for social information-which holds true for fish [209–213], Taking into account that *O*. *niloticus* is also known for its elaborate social behavior, it makes sense that we found *oxt* to be differentially expressed between subordinate and dominant males. Which components of submissive and dominant behavior oxytocin influences in Nile tilapia still remains to be elucidated.

Crhbp (corticotropin releasing hormone binding protein) is part of the HPI (hypothalamus-pituitary-interrenal-axis) and thus stress response. It binds to Crh and related proteins and thus modulates their bioactivity and may even trigger downstream signaling on its own [101]. The downregulation of *crhbp* in the pituitaries of dominant males observed in the present study could therefore be a result of the well-known involvement of cortisol signaling in reproduction (for review see Milla *et al*. [214]). In a recent study, Castañeda Cortés *et al*. [215] observed that *crhb* expressionis crucial for high temperature induced masculinization of female medaka embryos and an upstream regulator of steroidogenesis related genes like *cyp11c1*, *hsd11b2* and *cyp19a1* which were under differential regulation also in the present study. In social cichlids, elevated cortisol levels can be associated with subordinate rank [216] or with periods of social change [14, 217, 218]. Although recent findings in zebrafish suggest that cortisol can also act as a promoter of spermatogenesis [219], elevated cortisol levels in fish are generally agreed to inhibit reproduction (for review see Rousseau *et al*. [220]). Considering the stable hierarchies in which our experimental animals had been living, downregulation of genes from the stress axis in d-males thus reflects their well-established social rank and active reproduction.

### RT-qPCR validates RNA-Seq results

The testis expressions of 15 genes were examined with RT-qPCR to validate the results of RNA sequencing (Fig 6). In order to add more statistical power to the experiment, four additional fish (two of each social rank) were used together with the eight fish from RNA-Seq. The additional animals were assigned ranks based on their color, behavior and GSI. This strategy allowed us to make well-grounded predictions which genes were differentially expressed in fish based on their social status. In conclusion, both RNA-Seq and RT-qPCR yielded very similar results for the genes in question. For 12 out of 15 genes both methods yielded the same result. Seven genes were upregulated in the testes of d-males and for five genes no difference in expression was found. Only three genes were assessed differently by the two methods. The Gene encoding the androgen receptor *ar* was significantly upregulated in the testes of d-males according to RNA-Seq but RT-qPCR merely showed a trend in this direction, which was however not significant. In the case of *ctnnb2*, RNA-Seq analysis did not find differential expression between the social groups but RT-qPCR showed a significant downregulation in the testes of dominant males. The third case of discrepancy between the two methods was that of *amh*, while there was no differential regulation according to RNA-Seq, in the validation experiment by RT-qPCR, d-males had significantly lower levels of *amh* mRNA. The current annotation of the tilapia genome comprises two transcripts from a single *amh* gene on LG23. Close inspection of our sequencing data in the present study revealed that only a single transcript can be detected and no differential splicing of the *amh* gene occurred, similarly to what was already found by Poonlapdecha *et al*. [221]. Since it is known for a number of tilapia strains, that multiple *amh* paralogues like *amhY* and *amhΔY* can exist which often function as important male sex determinators [158, 164, 165, 168, 169, 222], we must also reckon with possible additional *amh* gene copies in our Nile tilapia strain. Nevertheless, we have already shown earlier that an *amhΔY* copy is not present in our strain [28] and we have found through our transcriptome analysis presented in this study that the *amhY* specific SNP (C/T—Ser/Leu92; [158]) did never occur. Therefore, we conclude that the expression data we got (RNA-Seq and RT-qPCR) reflects the expression from one *amh* gene on LG23. The lack of splice variants and the lack of the additional *amhY* and *amhΔY* copies in our lab strain thus demonstrates the versatility of this gene in teleost reproduction again.

## Conclusion

In conclusion, our study provides valuable gene expression data from stable, long-term dominant and subordinate Nile tilapia. Earlier studies on the expression profile of dominance have often focused on the brain and carved out the importance of the components of the different social networks like the preoptic area or the social decision-making network. The intricate signaling networks of the teleost pituitary in contrast have just started to be unraveled in recent years. In the present work, we augment the knowledge base by contributing gene expression data for the pituitary and testis. This allowed us to strengthen the evidence for the involvement of steroidogenesis related genes, growth factors and gonadotropins in social dominance. In addition to the androgens, 11-KT and testosterone, also estrogen levels and the expressions of a number of estrogen responsive genes were up-regulated in dominant males. This underlines the importance of estrogen in male reproduction. Our dataset also opens the possibility to put genes into a new, social context (e.g., *igfbp*s, *ptger*, *gfra*). We could also show that social dominance is defined by a general stimulation of reproduction related gene expression in d-males, which was reflected in pathway enrichment analyses. In congruency with our histological observations, subordinate males do not lose their spermatogenic capacity, however. Neither did they show structural abnormalities in their testes, nor were previously described key players of spermatogenesis (e.g. *fshr* or *igf3*) differentially expressed between social ranks. Plasma levels for Fsh did not significantly differ between d- and s-males and *fshb* was even down-regulated in the pituitaries of dominant males. Lh plasma levels and *lhb* expression on the other hand correlated with long-term social dominance. Our study thus paves the road to the identification of new factors involved in controlling gonad development in highly social teleosts such as Nile tilapia.

## Supporting information

S1 Fig Ration of interstitial areas in dominant and subordinate tilapia. (PNG)

S1 Table Gene lists used for pathway enrichment analysis. (XLSX)

S2 Table RNA-Seq results. (XLSX)

S3 Table Functional annotation results. (XLSX)

S4 Table Calculation of differential expression from RT-qPCR data. (XLSX)
