# Supplementary figures and images for: Transcriptomes of testis and pituitary from male Nile tilapia (O. niloticus L.) in the context of social status
Source: PLoS One. 2022 May 11;17(5):e0268140. doi: 10.1371/journal.pone.0268140 (PMC9094562; doi:10.1371/journal.pone.0268140)

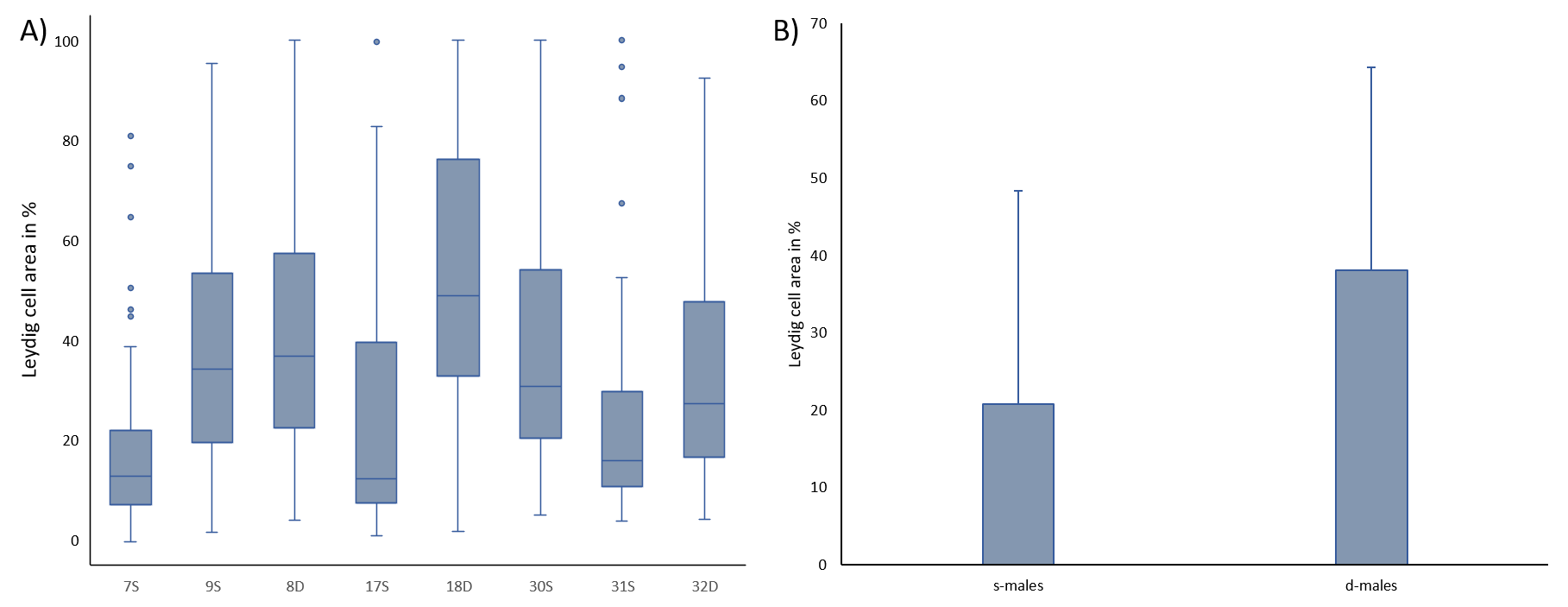

Supplement: S1 Fig — (PNG) [file pone.0268140.s001.png]
